# Impaired neuronal maturation of hippocampal neural progenitor cells in mice lacking CRAF

**DOI:** 10.1371/journal.pone.0192067

**Published:** 2018-03-28

**Authors:** Verena Pfeiffer, Rudolf Götz, Guadelupe Camarero, Helmut Heinsen, Robert Blum, Ulf Rüdiger Rapp

**Affiliations:** 1 University of Würzburg, Institute of Anatomy and Cell Biology, Koellikerstraße 6, Würzburg, Germany; 2 University of Würzburg, Institute for Medical Radiation and Cell Research (MSZ), Versbacher Strasse 5, Würzburg, Germany; 3 Institute for Clinical Neurobiology, University Hospital Würzburg, Versbacher Strasse 5, Würzburg, Germany; 4 University of Würzburg, Department of Psychiatry, Psychosomatics and Psychotherapy, Margarethe-Höppel-Platz 1, Würzburg, Germany; 5 Universidade de Sao Paulo Faculdade de Medicina, Pathology—LIM 44 Sao Paulo, SP, Brazil; 6 Department of Lung Development and Remodeling, Max Planck Institute for Heart and Lung Research, Parkstr.1, Bad Nauheim, Germany; Nathan S Kline Institute, UNITED STATES

## Abstract

RAF kinases are major constituents of the mitogen activated signaling pathway, regulating cell proliferation, differentiation and cell survival of many cell types, including neurons. In mammals, the family of RAF proteins consists of three members, ARAF, BRAF, and CRAF. Ablation of CRAF kinase in inbred mouse strains causes major developmental defects during fetal growth and embryonic or perinatal lethality. Heterozygous germline mutations in CRAF result in Noonan syndrome, which is characterized by neurocognitive impairment that may involve hippocampal physiology. The role of CRAF signaling during hippocampal development and generation of new postnatal hippocampal granule neurons has not been examined and may provide novel insight into the cause of hippocampal dysfunction in Noonan syndrome. In this study, by crossing CRAF-deficiency to CD-1 outbred mice, a CRAF mouse model was established which enabled us to investigate the interplay of neural progenitor proliferation and postmitotic differentiation during adult neurogenesis in the hippocampus. Albeit the general morphology of the hippocampus was unchanged, CRAF-deficient mice displayed smaller granule cell layer (GCL) volume at postnatal day 30 (P30). In CRAF-deficient mice a substantial number of abnormal, chromophilic, fast dividing cells were found in the subgranular zone (SGZ) and hilus of the dentate gyrus (DG), indicating that CRAF signaling contributes to hippocampal neural progenitor proliferation. CRAF-deficient neural progenitor cells showed an increased cell death rate and reduced neuronal maturation. These results indicate that CRAF function affects postmitotic neural cell differentiation and points to a critical role of CRAF-dependent growth factor signaling pathway in the postmitotic development of adult-born neurons.

## Introduction

Rapidly accelerated fibrosarcoma (RAF) kinases are a central part of the classical growth factor signaling cascade, the RAS-RAF-MEK-ERK signaling pathway. This signaling cascade regulates proliferation, differentiation, survival and apoptosis of many cell types [1]. RAF was discovered as a cancer gene (v-raf) [2] and subsequent studies identified three v-raf homologs in mammals, the RAF kinases ARAF [3], BRAF [4] and CRAF [5]. RAF proteins are activated by GTP-bound RAS, which binds RAF in its activated state and translocates it to the plasma membrane. Dimerization of RAF proteins leads to a complete RAF protein activation [6–12] and triggers the phosphorylation of the downstream signaling cascade [13]. The physiologic role of RAF kinases has been evaluated in mouse models. Ablation of BRAF causes embryonic death around embryonic day (E) 10.5–12.5 [14]. During fetal growth and development of inbred mice, inactivation of CRAF is lethal [15, 16]. In contrast, when crossed into an outbred genetic background, embryonic lethality was reduced, but the newborn CRAF-deficient pups dies within a few hours after birth [15, 17]. ARAF deficient mice survive up to postnatal day 21 (P21) [18]. Heterozygous germline mutations in CRAF have been discovered in Noonan syndrome, a developmental disorder characterized by pleiotropic phenotypes, including neurocognitive impairment, short stature and craniofacial malformations [19–21]. Mutations in CRAF account for approximately 5% of cases of Noonan syndrome, and the majority of these are gain-of function mutations encoding a kinase with enhanced MEK-ERK activity. A mouse model for one of these activating CRAF mutations, CRAF (L613V) displays, like patients with Noonan syndrome, eccentric cardiac hypertrophy, short stature and craniofacial dysmorphia but impairment in brain function were not investigated [22]. The pathogenic mechanism of Noonan-associated CRAF mutations with impaired kinase activity and reduced ERK phosphorylation is not known. Notably, Noonan syndrome patients show cognitive impairement and memory problems, including a reduced performance in hippocampus-dependent memory tasks [23]. In the hippocampus, there is a pool of neural progenitor cells located in the tertiary dentate gyrus matrix of the presumptive subgranular zone of the dentate gyrus [24]. In the dentate gyrus of mice, the majority of the granule neurons are born in the first postnatal week and neural progenitor cells retain their neurogenic potential during adulthood [25]. Adult neurogenesis in the subgranular zone is one of the most striking form of structural plasticity in the adult nervous system and contributes to hippocampal function [25]. Erk1 and Erk2 activity were found to be essential for synaptic plasticity in the hippocampus [26]. Compound mice with knockout of Mapk3 (Erk1) and forebrain-specific ablation of Mapk1 (Erk2) displayed significantly fewer granule neurons and near complete loss of neural progenitors in the postnatal hippocampus which blocked the generation of granule cells later in development [27]. We have previously reported that mice with a conditional elimination of BRAF showed a reduced ability of hippocampal progenitor cells to differentiate into granule cell neurons [28]. Whether altered CRAF function affects postnatal development and neuronal physiology in the dentate gyrus has not been studied so far. Here, we describe an important role of CRAF in postnatal and young adult hippocampal neurogenesis. We show that postnatal mice lacking CRAF kinase exhibit strongly increased cell proliferation, cell-cycle abnormalities, failure of post-mitotic differentiation and enhanced apoptotic cell death in the dentate gyrus of the hippocampus. This shows that CRAF signaling has a specific impact on cell cycle regulation and differentiation in the early phase of postnatal hippocampal neurogenesis.

## Results

### Phenotype of CRAF-deficient mice

**Fig 1:**
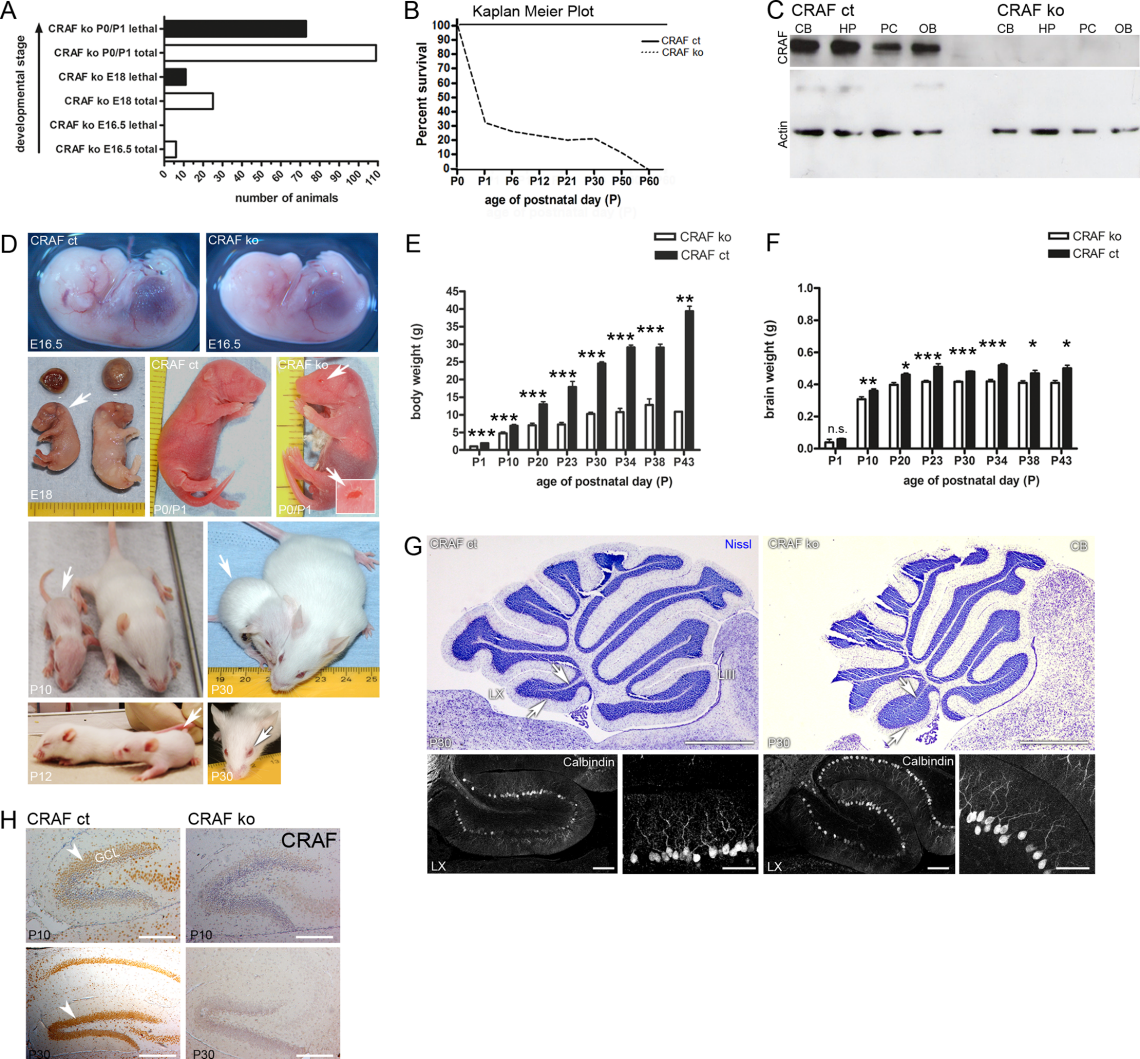
CRAF-deficiency in postnatal mice. (A) Lethality-analysis of CRAF ko embryos around birth (embryonic day 16.5, E16.5-newborn mice at postnatal day 0/1, P0/P1). White bars show total numbers of CRAF ko animals at different developmental stages (E16.5-P0/P1). Dark bars indicate the number of dead mice. No lethal CRAF ko embryos could be observed at E16.5. CRAF ko (E16.5 total), n = 6 (from n = 39 littermates); CRAF ko (E18 total), n = 25 (from n = 145 littermates); CRAF ko (E18 lethal), n = 11, indicating 47% lethality of CRAF ko at E18; CRAF ko (P0/P1 total), n = 109 (from n = 873 littermates); CRAF ko (P0/P1 lethal), n = 73 indicating 67% lethality of CRAF ko at P0/P1. (B) Kaplan-Meier survival curve of postnatal CRAF ct (dark line) and CRAF ko (pointed line) mice. Mice were daily monitored. CRAF ct, n = 763; CRAF ko, n = 110. (C) Western blot analysis of CRAF (C-20) expression in dissected brain areas (hp, hippocampus; pc, prefrontal cortex; cb, cerebellum; bo, olfactory bulb) of postnatal (P30) CRAF^+/+^ control (CRAF ct) and CRAF-deficient (CRAF^-/-^, CRAF ko) mice. ß-actin serves as loading control. (D) Phenotypic analysis of CRAF ko mice compared to CRAF ct siblings. (Upper line) CRAF ko embryos (right panel) are anyhow smaller and appear more shiny compared to CRAF ct siblings (left panel) at E16.5. (2^nd^ line) From E18 (left panel) on CRAF ko animals (white arrow) are clearly discernible due to their reduced body size compared to CRAF ct siblings. The placenta is shown on top of animals. (2^nd^ line, right panel) CRAF ko mice were later born with open eyes (white arrow and Inset). (3^rd^ line left) Postnatal CRAF ko mice are strongly reduced in body size at the age of P10 (white arrow) with less hair and pink skin color shining through the coat. (3^rd^ line right) Postnatal CRAF ko mice at the age of P30 showed reduced body size (white arrow) compared to CRAF ct siblings (right). (Lower panel left) Postnatal CRAF ko mice with eye open at birth (EOB) phenotype at the age of P12 compared to CRAF ct sibling with closed eyes. (Lower panel right) CRAF ko mouse at the age of P30 with eyelid defect (white arrow). (E) Quantitative body weight analysis of CRAF ct (dark bars) and CRAF ko (white bars) siblings (n = 234) from postnatal day P1 until P43. Data are mean ± s.e.m.; n = 234. Significant differences are shown in p-value p< 0.05 (*), p< 0.01 (**), p< 0.001 (***). (F) Quantitative brain weight analysis of CRAF ct (dark bars) and CRAF ko (white bars) siblings (n = 234) from postnatal day P1 until P43. Data are mean ± s.e.m.; significant differences are shown in p-value p< 0.05 (*), p< 0.01 (**), p< 0.001 (***). (G) Cerebellar abnormalities in postnatal CRAF ko mice. (Upper panel) Representative sagittal sections of Nissl stained cerebellum of CRAF ct (left) and CRAF ko (right) mice at the age of P30. White arrows in CRAF ko (upper panel right) indicate alterations in lobule X (LX) related to reduced length, failure of proper build central core stream and sulcus compared to CRAF ct (left, white arrows). Also LIII appeared strongly reduced in size in CRAF ko (upper panel right) compared to CRAF ct (left). Scale bars = 1mm. (Lower panel) Representative sagittal sections of Calbindin stained cerebellum LX of CRAF ct (left) and CRAF ko (right) mice at the age of P30. Calbindin-stained Purkinje cell neurons of CRAF ko mice are irregular located in LX. Scale bars = 60μm. Higher magnification of Calbindin-staining reveals elongated and less arborescent dendrite formation of Purkinje cell neurons through the molecular layer. Scale bars = 60μm. (H) Analysis of CRAF expression by immunohistochemistry. Representative sagittal sections of P10 (upper panels) and P30 (lower panels) CRAF ct (left panels) and CRAF ko (right panels) hippocampus stained for CRAF (C-20). White arrowheads indicate CRAF stained cell body of granule neurons in the granule cell layer (GCL) of the hippocampal dentate gyrus in CRAF ct (left panels). CRAF ko sections exhibit no CRAF-expression (right panel). Sections were counterstained with hematoxylin. Scale bar = 50μm.

Genetic ablation of CRAF in a variety of inbred mouse strains caused gross abnormalities and death before birth [16, 17]. When crossed to an outbred genetic background [15, 17], growth-retarded *craf* ^*-/-*^ pups were born alive but died within a few hours of birth. Here, we crossed heterozygous *craf* ^*+/-*^ mice harboring a *craf* null-allele on a 129Ola/C57BL6 background [17] for two generations to a CD-1 or MF1 outbred strain to reach a CD-1 or MF1 fraction of ≥75%. We obtained higher numbers of living *craf* ^*-/-*^ animals from the CD-1 background; thus, all subsequent experiments were performed with *craf* ^*-/-*^ animals and their littermates generated by CRAF^+/-^ CD-1 intercrosses. CRAF-deficient mice from heterozygous intercrosses (CRAF^+/-^ x CRAF^+/-^) were born below the expected *Mendelian ratio* (n = 873 newborn mice; n = 110 CRAF^-/-^; 13% CRAF^-/-^ littermates). The mortality rate was high around birth (n = 74/110; 67%; Fig 1A and 1B). The majority of the mice that had survived during this critical perinatal period were still alive at day P30; three mice lived up to 55 days of age (Fig 1B). Western analysis using an antibody targeted against the N-terminus of CRAF confirmed expression of CRAF protein with a relative molecular weight of 74 kDa in the cerebellum, hippocampus, cortex and olfactory bulb of postnatal CRAF ct mice (n>10, P10-P30). CRAF immunoreactivity was lost in corresponding protein samples obtained from CRAF^-/-^ mice (Fig 1C) and we could not find drastic changes concerning ERK1/2 phosphorylation in protein samples harvested from the cerebellum, the hippocampus, prefrontal cortex and olfactory bulb (S7 Fig). This observation is in line with earlier studies that already described comparable pERK levels in CRAF ko mice in comparison to wild-type mice [16, 17]. At embryonic day E16.5 CRAF-deficient embryos did not show gross alterations in body size compared to control siblings (CRAF^+/+^) (Fig 1D); no dead CRAF^-/-^ embryo was observed at E16.5 (n = 0/6; n = 39 analyzed embryos at E16.5; Fig 1A). In contrast, CRAF-deficient animals shortly before birth (E18) were reduced in size (Fig 1D) and showed a mortality rate of 44% (E18; n = 11/25) (Fig 1A). Staining for activated-caspase 3 at late embryonic (E18) and early postnatal (P0/P1) stages revealed a ~20 fold increase in apoptotic cells in the lung (E18, n = 4, p<0.001; P0/P1, n = 6, p<0.0001) and ~8 fold increase in apoptotic cells in the liver (E18, n = 4, p<0.001; P0/P1 liver n.s.) of CRAF^-/-^ pups compared to control. Newborn CRAF-deficient mice were phenotypically conspicuous by the *eye open at birth* (EOB) [29] phenotype [15] (Fig 1D; P0/P1, P12 white arrows and S1A Fig) and appeared brighter by a pale skin color (Fig 1D) compared to control (CRAF^+/+^) siblings. Postnatal CRAF^-/-^ mice at P10 appeared less hairy by the fact that the skin color shines through the coat [15] (Fig 1D, P10, white arrow). The EOB-phenotype observed in CRAF^-/-^ at P0/P1 (Fig 1D and S1A Fig) leads to eyelid closure defects at later postnatal stage (Fig 1D P30 white arrow and S1B Fig). Body weight analysis of postnatal CRAF^-/-^ and CRAF^+/+^ mice revealed a significant reduction in body weight of CRAF^-/-^ already after birth (P1) (Fig 1E and S1C Fig). At P30 CRAF^-/-^ littermates were much smaller (Fig 1D, P30 white arrow) and showed a remarkable 2.4 fold reduction in body weight (CRAF^-/-^ 10,2g; CRAF^+/+^ 24,55g) compared to CRAF^+/+^ siblings (Fig 1D, P30). The brain weight of CRAF^-/-^ was significantly decreased from P10 onwards (Fig 1F).

### Psychophysical tests

Behavioral observations with pronounced alterations in postnatal day P30 CRAF^-/-^ mice were monitored when mice were placed on cage top. Here, CRAF^-/-^ mice were impaired to raise up their body catching the cage top with front limbs only (S2A Fig), whereas CRAF^+/+^ siblings performed gymnastics by temporarily hanging with only one leg on headlong (S2A Fig). Further tests show that postnatal day P30 CRAF^-/-^ mice were impaired in the ability to balance on a small stick and fall down during less than two seconds (n = 3) (S2B Fig), while CRAF^+/+^ mice move from one end of the rod to the other, hanging in between headlong before changing the orientation (S2B Fig). Behavior analysis focused on motor coordination was conducted with postnatal day P30 CRAF^-/-^ and CRAF^+/+^ siblings on a Rotarod (n = 3) (S2C Fig). The high mortality rate and the observed phenotypical changes limited the possibilities for a detailed behavioral and psychophysical analysis of the CRAF^-/-^ animals for practical and ethical reasons. For the phenotypical description (Rotarod experiment), we excluded CRAF^-/-^ mice where one or both eyelids were closed. We investigated only mice which showed a high exploratory activity in their homecage and with both eyelids open (S1B Fig). The counted time spent on the Rotarod showed no significant differences between CRAF^-/-^ and CRAF^+/+^ control mice (S2D Fig).

### Morphological alterations in the cerebellum

Postnatal CRAF control brain sections showed an expression of CRAF in cerebellar Purkinje cells that was absent in CRAF-deficient mice (S4A–S4C Fig). Histological analysis of Nissl stained sagittal brain sections from postnatal day P10 and P30 CRAF^-/-^ and CRAF^+/+^ mice revealed no gross alteration with the exception of the cerebellum (Fig 1G and S3 Fig white arrowheads). CRAF^-/-^ mice showed morphological alterations in lobule III (LIII) and lobule X (LX) [30] (Fig 1G). Both structures appeared shorter in postnatal CRAF^-/-^ mice with a strongly reduced medullary layer and especially LX appeared compressed (Fig 1G and S3 Fig white arrowheads) with an exaggerated multilayering of calbindin-positive Purkinje cell cell-bodies (Fig 1G). Furthermore CRAF^-/-^ Purkinje cells exposed elongated primary dendrites without any general espalier fruit design (Fig 1G). Similar results have been already observed in Purkinje cells lacking BRAF kinase [28].

### Hippocampal neurogenesis in young CRAF-deficient mice

**Fig 2:**
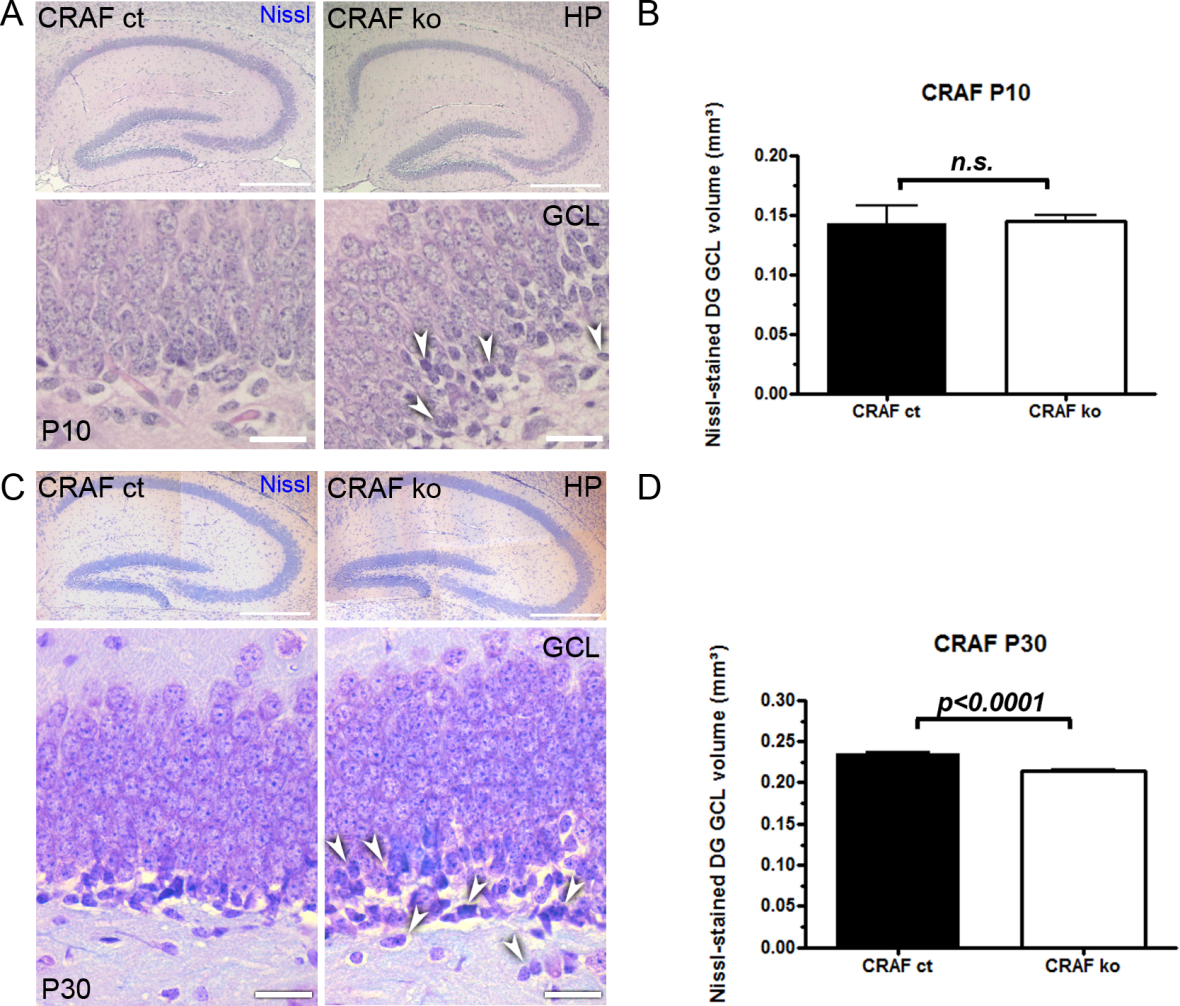
Decreased DG GCL volume of postnatal CRAF ko mice. (A) Immuno-histological analysis of Nissl stained sagittal brain sections of CRAF ct and CRAF ko hippocampus at P10. (Upper panel) Representative brain section of CRAF ko (right) reveals no general morphological alterations of the hippocampus compared to CRAF ct (left). Scale bar = 100μm. (Lower panel) Higher magnification of CRAF ct (left panel) and CRAF ko (right panel) dentate gyrus granule cell layer (GCL). CRAF ko slices show clusters of darkly stained Nissl cells in the subgranular zone of the dentate gyrus GCL (white arrowheads). Scale bar = 50μm. (B) Quantitative analysis of Nissl-stained dentate gyrus (DG) GCL of CRAF ct (dark bar) and CRAF ko (white bar) at P10 (n = 8). Data are mean ± s.e.m.; no significant differences were detected. (C) (Upper panel) Representative sagittal sections of P30 hippocampus of CRAF ct (left panel) and CRAF ko (right panel) stained with Nissl. CRAF-deficient mice (right panel) display no alterations in the general morphology of the hippocampus. Scale bar = 100μm. (Lower panel) Higher magnification of CRAF ct (left panel) and CRAF ko (right panel) dentate gyrus granule cell layer (GCL). CRAF ko slices show clusters of darkly stained, chromophilic cells in the subgranular zone and hilus of the dentate gyrus GCL (white arrowheads). Scale bar = 50μm. (D) Volume of Nissl-stained dentate gyrus (DG) GCL of CRAF ct (dark bar) and CRAF ko (white bar) at P30 (n = 30). Data are mean ± s.e.m.; significant differences are shown in p-value p< 0.0001.

We examined the distribution of CRAF in the dentate gyrus of postnatal and young adult mice by immunohistochemistry. Postnatal CRAF control brain sections showed an abundant expression of CRAF in pyramidal cells of the Cornu ammonis (CA) region, in granule cell neurons of the dentate gyrus and weak expression in cells located in the subgranular zone. CRAF-deficient mice showed no CRAF immunoreactivity, as expected (Fig 1H). We could not find gross alterations in the general morphology of the hippocampus of CRAF^-/-^ mice at postnatal day P10 (Fig 2A) (n = 8) and P30 (n = 30) (Fig 2C) compared to CRAF^+/+^ control siblings. Quantitative analysis of Nissl stained dentate gyrus GCL volume revealed no difference between CRAF^-/-^ and control mice at postnatal day P10 (n = 8) (Fig 2B), indicating that the generation of granule cell neurons from the tertiary dentate matrix is not affected by the loss of CRAF. However, at postnatal day P30, CRAF^-/-^ dentate gyrus Nissl-stained GCL volume was significantly decreased by ~10% compared to CRAF^+/+^ control siblings (Fig 2D). This observation indicates that CRAF signaling seems to play a central role in the development of the hippocampus starting at around day 10 of age.

**Fig 3:**
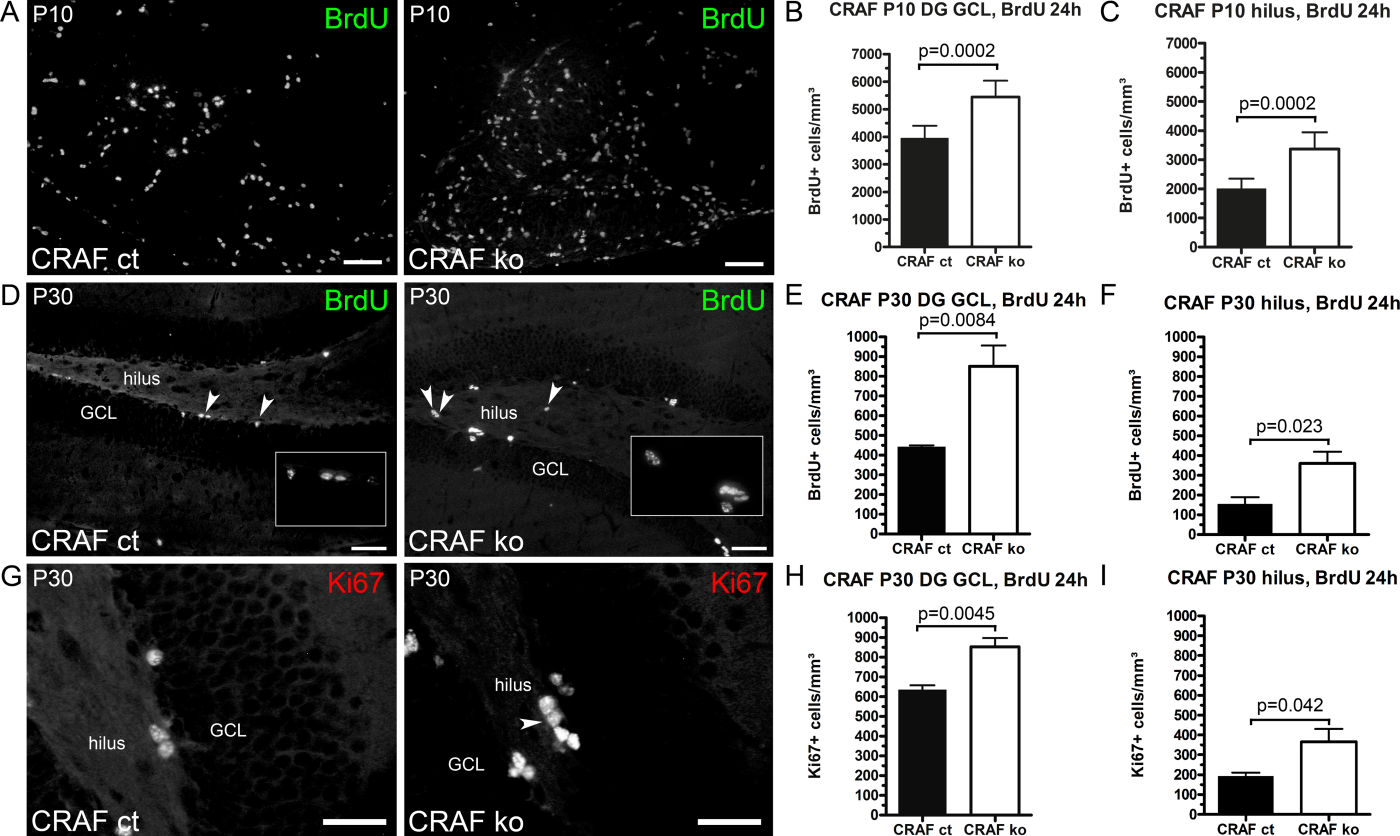
Increased NPC proliferation in the DG GCL and hilus of postnatal CRAF ko mice. (A) Immuno-histological analysis of BrdU stained sagittal brain sections of CRAF ct and CRAF ko hippocampus at P10 24h after a single BrdU application. Representative brain sections of CRAF ct (left panel) and CRAF ko (right panel) show higher amounts of proliferative BrdU-labelled precursor cells in the dentate gyrus of CRAF ko. Scale bar = 50μm. (B) Quantitative analysis of BrdU-stained dentate gyrus (DG) GCL of CRAF ct (dark bar) and CRAF ko (white bar) at P10 (n = 7) 24h after a single BrdU application. Data are mean ± s.e.m.; significant differences are shown in p-value p = 0.002. (C) Quantitative analysis of BrdU-stained hilus of CRAF ct (dark bar) and CRAF ko (white bar) at P10 (n = 7) 24h after a single BrdU application. Data are mean ± s.e.m.; significant differences are shown in p-value p = 0.0002. (D) Immuno-histological analysis of BrdU stained sagittal brain sections of CRAF ct and CRAF ko hippocampus at P30 24h after a single BrdU application. Representative brain sections of CRAF ct (left panel) and CRAF ko (right panel) show higher amounts of proliferative BrdU-labelled precursor cells in the hippocampus of CRAF ko. White arrowheads indicate the localization of BrdU labelled cells in the DG GCL of CRAF ct (left panel) and in the DG GCL and hilus of CRAF ko (right panel). Scale bar = 50μm. (E) Number of of BrdU-stained dentate gyrus (DG) GCL of CRAF ct (dark bar) and CRAF ko (white bar) at P30 (n = 4) 24h after a single BrdU application. Data are mean ± s.e.m.; significant differences are shown in p-value p = 0.0084. (F) Number of of BrdU-stained hilus of CRAF ct (dark bar) and CRAF ko (white bar) at P30 (n = 4) 24h after a single BrdU application. Data are mean ± s.e.m.; significant differences are shown in p-value p = 0.023. (G) Immuno-histological analysis of Ki67 stained sagittal brain sections of CRAF ct and CRAF ko hippocampus at P30. Representative brain sections of CRAF ct (left panel) and CRAF ko (right panel) show higher amounts of proliferative Ki67 positive precursor cells in the hippocampus of CRAF ko. White arrowhead indicates the localization of many Ki67 positive cells in the hilus of CRAF ko (right panel). Scale bar = 50μm. (H) Quantitative analysis of Ki67-stained dentate gyrus (DG) GCL of CRAF ct (dark bar) and CRAF ko (white bar) at P30 (n = 4). Data are mean ± s.e.m.; significant differences are shown in p-value p = 0.0045. (I) Quantitative analysis of Ki67-stained hilus of CRAF ct (dark bar) and CRAF ko (white bar) at P30 (n = 4). Data are mean ± s.e.m.; significant differences are shown in p-value p = 0.042.

In Nissl-stained sections, we detected a remarkably increased number of smaller, oval, chromophilic cells in the basal parts of granule cell layer and the hippocampal hilus of CRAF^-/-^ mice at P10 and P30 (Fig 2A and 2C white arrowheads). To examine the hypothesis that the darkly stained cells in the dentate gyrus of CRAF^-/-^ mice (Fig 2A and 2C white arrowheads) represent proliferating precursor cells as suggested by Palmer et al. [31] and Nilsson et al. [32], we performed BrdU-labelling experiments. Mice were injected with a single dose of BrdU at postnatal day P10 or P30 and were sacrificed 24h later. Immunohistological analysis of BrdU-labelled hippocampal sections revealed a significant increase in proliferating neural progenitor cells (NPCs) in the GCL and hilus of CRAF^-/-^ at P10 (Fig 3A–3C; DG GCL Δ~28%, p = 0.002; hilus Δ~41%, p = 0.0002; n = 7) and P30 (Fig 3D–3F; DG GCL Δ~48%, p = 0.0084; hilus Δ~57%, p = 0.023; n = 4). To further investigate the proliferation of NPCs in the dentate gyrus of P30 mice, we quantified the number of Ki67-positive cells. Ki67 is expressed in dividing cells for the entire duration of their mitotic process [33, 34]. We observed an increased number of Ki67+ cells in the GCL (Δ~26%, p = 0.0045 n = 4) and hilus **(**Δ~48%, p = 0.042, n = 4) of CRAF-deficient mice (Fig 3G–3I).

**Fig 4:**
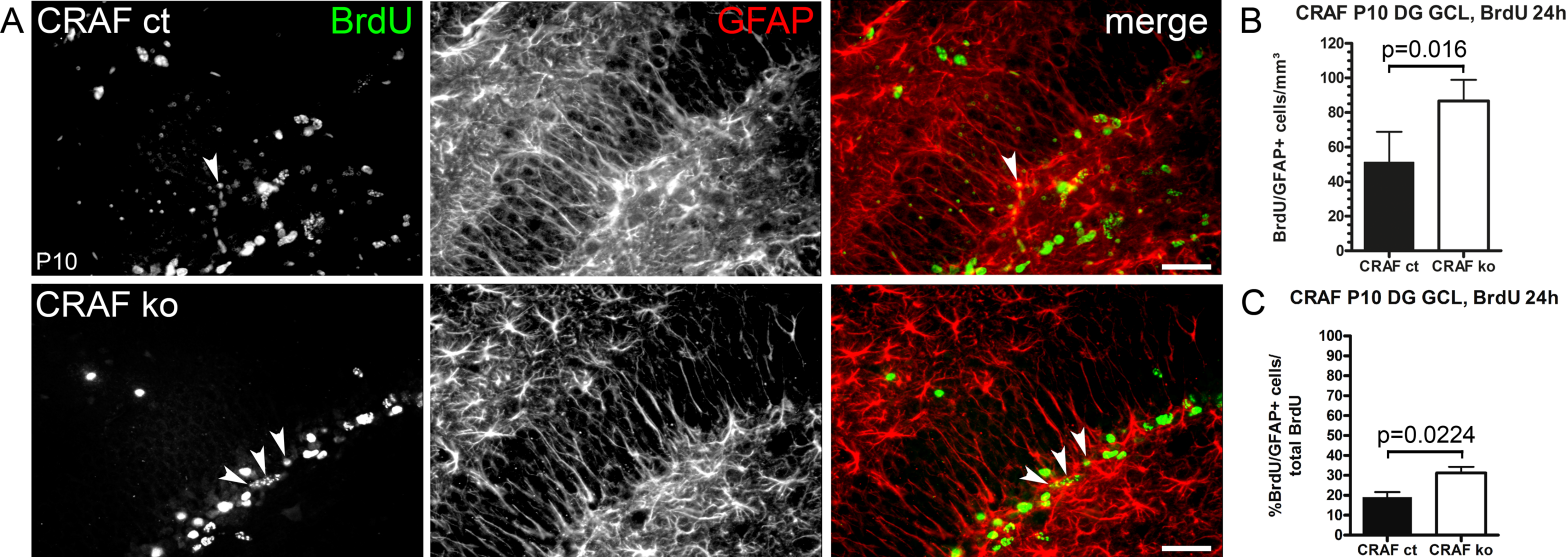
Increased numbers of BrdU-labelled NPCs with radial GFAP-positive processes in the DG GCL of CRAF ko at P10. (A) Immuno-histological analysis of BrdU (green) and the astrocytic marker GFAP (red) stained sagittal brain sections of CRAF ct and CRAF ko hippocampus at P10 24h after a single BrdU application. Representative brain sections of CRAF ct (upper panel) and CRAF ko (lower panel) show BrdU-labelled cells (green) colocalizing with GFAP-positive radial processes (red) (merge, white arrowheads). Scale bar = 50μm. (B) Quantitative analysis of BrdU/GFAP-stained neural precursor cells in the dentate gyrus (DG) GCL of CRAF ct (dark bar) and CRAF ko (white bar) at P10 (n = 4). The graph shows BrdU-labelled cells that colocalize with GFAP in radial processes 24h after a single BrdU application. Data are mean ± s.e.m.; significant differences are shown in p-value p = 0.016. (C) BrdU/GFAP positive cells as a fraction of BrdU-labelled cells in the dentate gyrus (DG) GCL of CRAF ct (dark bar) and CRAF ko (white bar) at P10 (n = 4). Data are mean ± s.e.m.; significant differences are shown in p-value p = 0.0224.

Granule cells are descendants of glial fibrillary acidic protein (GFAP)-expressing precursors within the dentate gyrus neurogenic niche that originate from radial glia [35–38]. Therefore, we wanted to determine whether the BrdU-positive cells are immature precursor cells with radial morphology that are not yet committed to neuronal development [35–40]. Double immune staining for BrdU and GFAP demonstrated an increased density of GFAP and BrdU-double-positive radial glia-like cells (rA) in the dentate gyrus GCL of CRAF^-/-^ at P10 (Δ~39% compared to CRAF^+/+^ control siblings (Fig 4A and 4B; p = 0.016, n = 4) and an enhanced fraction of BrdU and GFAP-double positive cells in the subgranular region of CRAF ko brains (Δ~12% compared to CRAF^+/+^ control siblings, Fig 4C, p = 0.0224, n = 4).

The number of cells double-positive for GFAP and Nestin, another marker that labels radial glia-like neuronal progenitor cells, was increased by 59% in the subgranular zone and hilus of CRAF-deficient mice (n = 3). This indicates that CRAF-deficient mice carry a higher number of GFAP-positive, Nestin-positive neural precursors in their postnatal dentate gyrus. In order to investigate whether the increased number of BrdU-labeled radial astrocytes persisted for longer periods of time, we applied a single pulse of BrdU at P23 and analyzed the brains at P35. The results showed a ~24% increase of BrdU- and GFAP-positive radial astrocytes in CRAF ko brains (p = 0.0009, n = 6; S5 Fig). We noted that the majority of BrdU- and GFAP-positive cells showed the typical morphology of radial glia. GFAP-expressing precursors in the SGZ of the dentate gyrus fall into two morphological classes, radial and horizontal, and both populations display NPC features [40, 41]. The fraction of horizontal astrocytes, albeit much smaller than the radial ones, was also significantly increased in the absence of CRAF (S5 Fig). These results indicate that inactivation of CRAF results in a higher number of proliferating progenitor cells in the subgranular zone and hilus.

**Fig 5:**
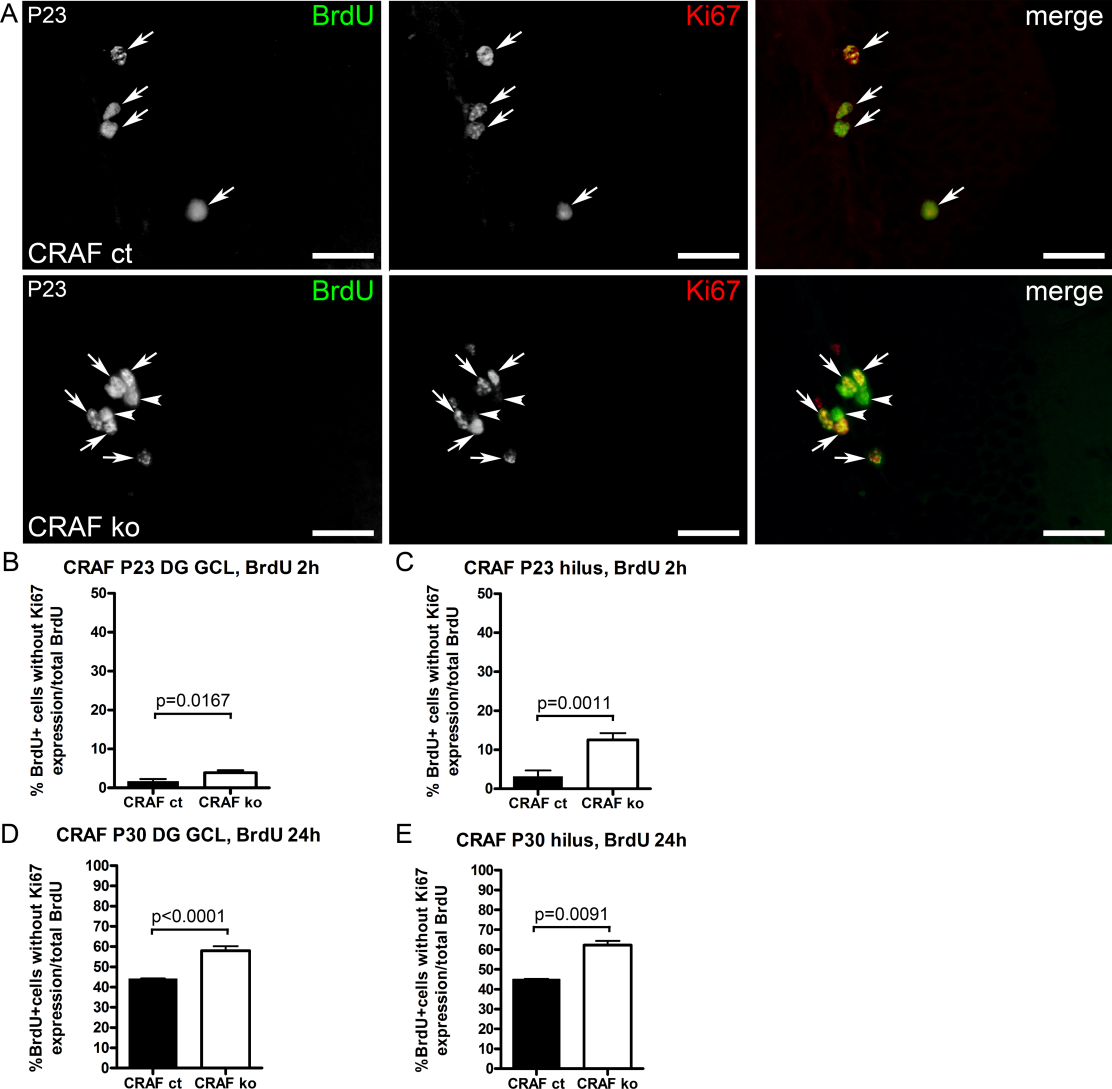
Cell cycle abnormalities in BrdU-labelled NPCs of postnatal CRAF ko mice. (A) Immuno-histological analysis of BrdU (green) and Ki67 (red) stained sagittal brain sections of CRAF ct and CRAF ko hippocampus at P23 2h after a single BrdU application. Representative brain sections of CRAF ct (upper panel) and CRAF ko (lower panel) show BrdU-labelled cells (green) colocalizing with Ki67 (red) (merge, white arrows). White arrowheads indicate BrdU-labelled cells (green) of CRAF ko that lack any positive Ki67 (red) staining (lower panel). Scale bar = 50μm. (B) Quantitative analysis of BrdU/Ki67-stained proliferative precursor cells in the dentate gyrus (DG) GCL of CRAF ct (dark bar) and CRAF ko (white bar) at P23 (n = 5). The graph shows the fraction of BrdU-labelled cells that lack any positive Ki67 expression 2h after a single BrdU application. Data are mean ± s.e.m.; significant differences are shown in p-value p = 0.0167. (C) Quantitative analysis of BrdU/Ki67-stained proliferative precursor cells in the hilus of CRAF ct (dark bar) and CRAF ko (white bar) at P23 (n = 5). The graph shows the fraction of BrdU-labelled cells that lack any positive Ki67 expression 2h after a single BrdU application. Data are mean ± s.e.m.; significant differences are shown in p-value p = 0.0011. (D) Quantitative analysis of BrdU/Ki67-stained proliferative precursor cells in the dentate gyrus (DG) GCL of CRAF ct (dark bar) and CRAF ko (white bar) at P30 (n = 6). The graph shows the fraction of BrdU-labelled cells that lack any positive Ki67 expression 24h after a single BrdU application. Data are mean ± s.e.m.; significant differences are shown in p-value p<0.0001. (E) Quantitative analysis of BrdU/Ki67-stained proliferative precursor cells in the hilus of CRAF ct (dark bar) and CRAF ko (white bar) at P30 (n = 6). The graph shows the fraction of BrdU-labelled cells that lack any positive Ki67 expression 24h after a single BrdU application. Data are mean ± s.e.m.; significant differences are shown in p-value p = 0.0091.

**Fig 6:**
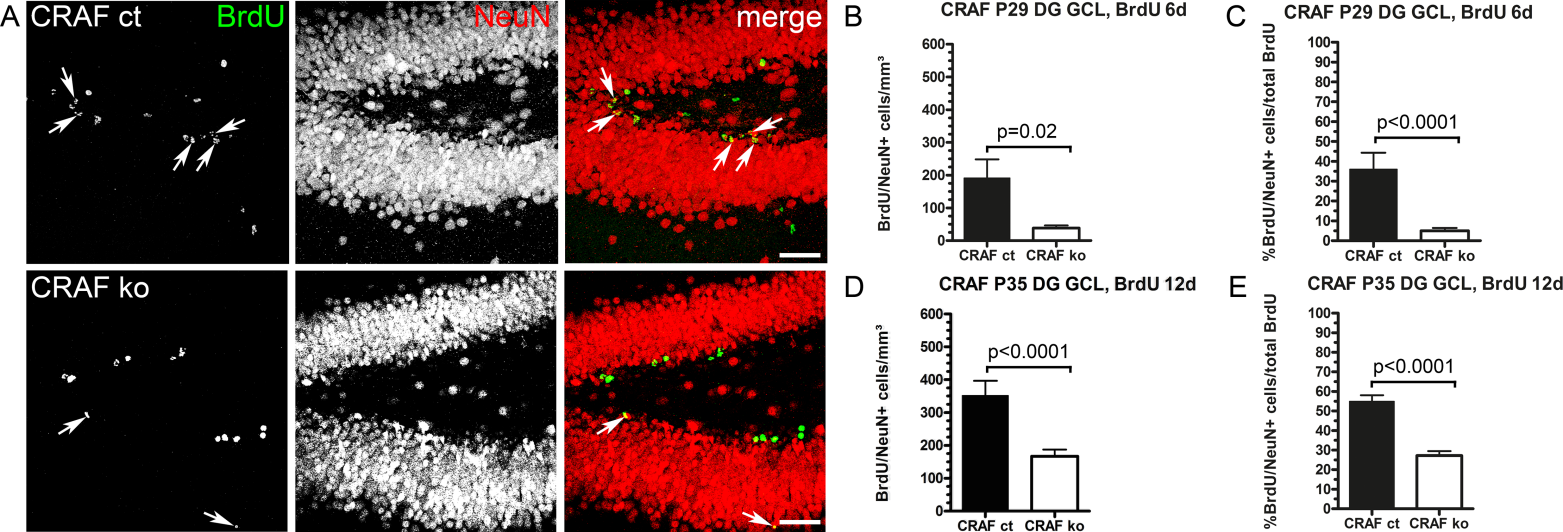
Impaired neuronal differentiation (maturation) of BrdU-labelled NPCs in the DG GCL of postnatal CRAF ko mice. (A) Immuno-histological analysis of BrdU (green) and the neuronal marker NeuN (red) stained sagittal brain sections of CRAF ct and CRAF ko hippocampus at P35 12 days after a single BrdU application. Representative brain sections of CRAF ct (upper panel) and CRAF ko (lower panel) show BrdU-labelled cells (green) colocalizing with NeuN (red) (merge, white arrows). Scale bar = 50μm. (B) Quantitative analysis of BrdU/NeuN-stained neural precursor cells with neuronal cell fate determination in the dentate gyrus (DG) GCL of CRAF ct (dark bar) and CRAF ko (white bar) at P29 (n = 6). The graph shows BrdU-labelled cells that colocalize with NeuN 6 days after a single BrdU application (P23→P29). Analysed cells were normalized with measured GCL Nissl-volume. Data are mean ± s.e.m.; significant differences are shown in p-value p = 0.02. (C) BrdU/NeuN positive cells as a fraction of BrdU-labelled cells in the dentate gyrus (DG) GCL of CRAF ct (dark bar) and CRAF ko (white bar) at P29 (n = 6) 6 days after a single BrdU application (P23→P29). Data are mean ± s.e.m.; significant differences are shown in p-value p<0.0001. (D) Quantitative analysis of BrdU/NeuN-stained neural precursor cells with neuronal cell fate determination in the dentate gyrus (DG) GCL of CRAF ct (dark bar) and CRAF ko (white bar) at P35 (n = 6). The graph shows BrdU-labelled cells that colocalize with NeuN 12 days after a single BrdU application (P23→P35). Data are mean ± s.e.m.; significant differences are shown in p-value p<0.0001. (E) BrdU/NeuN positive cells as a fraction of BrdU-labelled cells in the dentate gyrus (DG) GCL of CRAF ct (dark bar) and CRAF ko (white bar) at P35 (n = 6) 12 days after a single BrdU application (P23→P29). Data are mean ± s.e.m.; significant differences are shown in p-value p<0.0001.

To determine whether the increased number of proliferating cells in the dentate gyrus GCL (Fig 3B, 3E and 3H) and hilus (Fig 3C, 3F and 3I) of CRAF-deficient mice results from a shift in the fraction of progenitor cells that remain in the progenitor state instead of undergoing cell cycle exit, we determined cell cycle exit by examination of the fraction of cells dividing after pulse labelling with BrdU 2 or 24 hours earlier. We identified cells that had left the cell cycle as BrdU-positive cells that lack Ki67 expression. We detected a higher proportion of BrdU-labelled NPCs in the GCL and hilus that lacked Ki67 expression (DG GCL: 2h BrdU chase P23 Δ~56%, p = 0.0167, n = 5; 24h BrdU chase P30 Δ~24%, p<0.0001, n = 6; hilus: 2h BrdU chase P23 Δ~28% p = 0.0011, n = 5; 24h BrdU chase P30 Δ~28%, p = 0.0091, n = 6) (Fig 5A–5E). This finding indicates that the lack of CRAF signaling led to an increase in the fraction of progenitor cells which exit the cell cycle and/or which exhibit a shortened cell cycle. So far we have shown that CRAF-deficiency enhances NPC proliferation (Figs 3 and 4) and shortens cell cycle length of cell division (Fig 5). In order to elucidate the role of CRAF in neuronal cell fate determination and maturation, BrdU birthdating studies were conducted at different time points during postnatal development (P10, P23, P30) coupled with long chase periods of six and 12 days after a single application of BrdU. The hippocampus was stained for BrdU and NeuN to identify granule cells born at the time of BrdU application. After a chase period of six days after a single BrdU application, we observed significantly higher numbers of BrdU-labelled NPCs in the dentate gyrus GCL (Δ~28%, p = 0.007, n = 6) and hilus (Δ~49%, p = 0.0001, n = 6) of CRAF^-/-^ compared to CRAF^+/+^ control mice. In contrast, the number of BrdU and NeuN-positive cells was significantly reduced (Δ~86% 6 day chase, p<0.0001, n = 6; Δ~51% 12d chase, p<0.0001, n = 6) (Fig 6A, 6B and 6D). Furthermore, we observed a strong increase in neuronal differentiation of CRAF^-/-^ NPCs after a chase phase of 12 days compared to the six days chase phase data (Δ~82%) (Fig 6B–6E). This indicated that neuronal differentiation and maturation need more time in CRAF-deficient, compared to CRAF control mice (Fig 6A, 6C and 6E). These findings demonstrate that the ability of CRAF-deficient neural progenitor cells to differentiate into granule cell neurons is severely reduced and may explain the observed reduction in GCL volume of 30-day old CRAF-deficient brains.

### Increased apoptosis in the CRAF-deficient hippocampus

**Fig 7:**
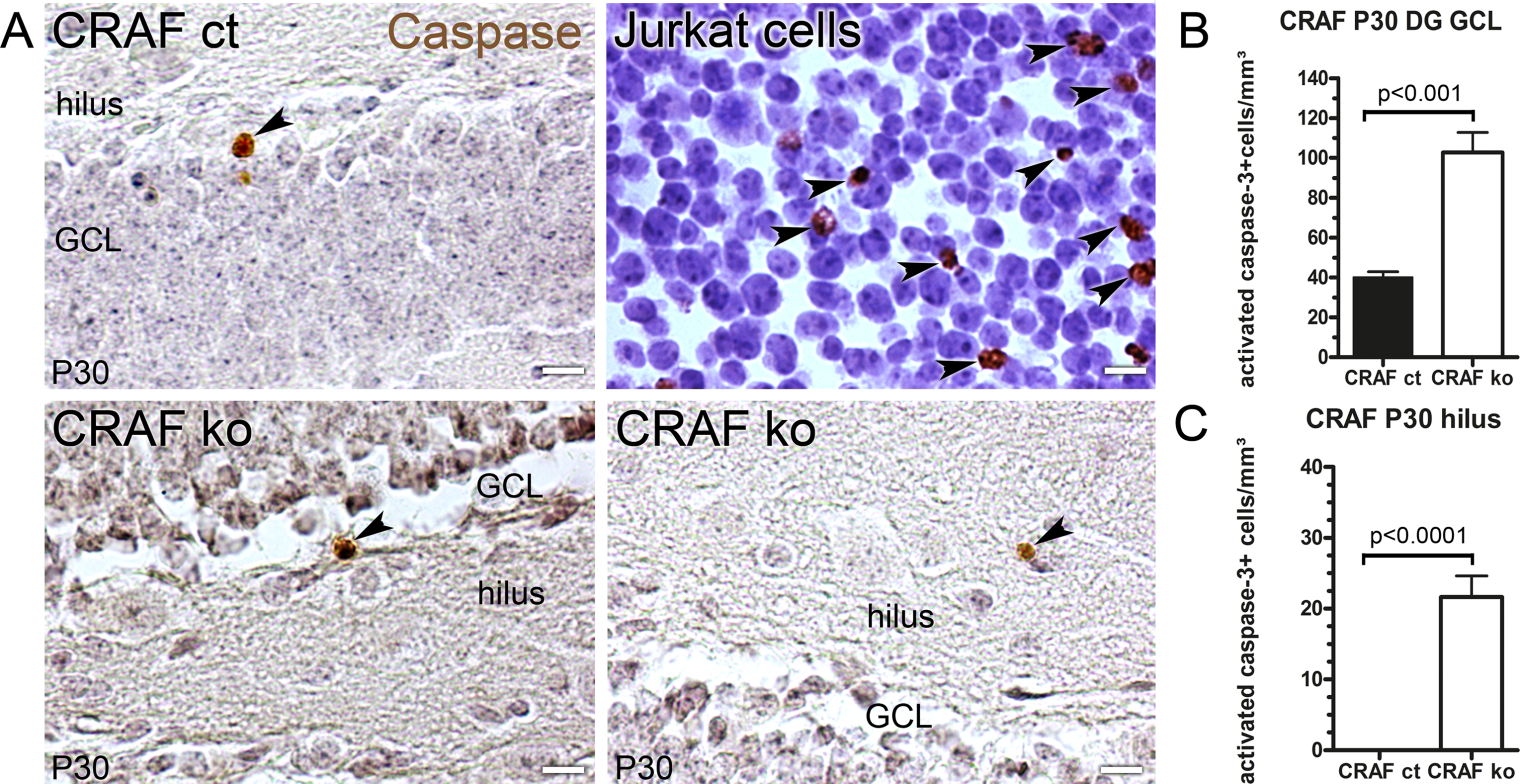
Cell fate analysis of postnatal P30 CRAF ko mice. A) Immuno-histological analysis of activated Caspase 3 (brown) stained sagittal brain sections of CRAF ct and CRAF ko hippocampus at P30. Tissue was counterstained with hematoxylin (grey-blue). Representative brain sections of CRAF ct (upper panel, left) and CRAF ko (lower panel). Etoposide-treated Jurkat cells (#8104, cell signalling) serve as positive control (upper line, right). Dark arrowheads indicate activated Caspase 3 positive stained cell nuclei. CRAF ko (lower panel) mice display higher amounts of activated caspase 3 positive cells in the GCL and hilus compared to CRAF ct (upper panel, left). Negative control was stained with secondary antibody only (shown in S1 Fig). Scale bar = 10μm. (B) Quantitative analysis of activated Caspase 3 positive cells in the dentate gyrus (DG) GCL of CRAF ct (dark bar) and CRAF ko (white bar) at P30. The graph shows activated Caspase 3 positive stained cells normalized with the GCL Nissl-volume. Data are mean ± s.e.m.; P30, n = 4. Significant differences are shown in p-value p<0.001. (C) Quantitative analysis of activated Caspase 3 positive cells in the dentate gyrus (DG) and hilus of CRAF ct (dark bar) and CRAF ko (white bar) at P30. The graph shows activated Caspase 3 positive stained cells normalized with the GCL Nissl-volume. Data are mean ± s.e.m.; P30, n = 4. Significant differences are shown in p-value p<0.0001.

The generation of granule cells in the dentate gyrus is regulated at the level of proliferation, differentiation and survival. The significant reduction in neuronal differentiation of CRAF^-/-^ NPCs in the dentate gyrus GCL compared to CRAF^+/+^ control mice (Fig 6) prompted us to investigate whether CRAF-deficiency is associated with changes in cellular survival, as was already reported by Wang et al. [42, 43]. In order to stain cells after induction of apoptosis, we immunolabelled hippocampal sections at P30 for activated caspase 3. We found a dramatic increase in the number of apoptotic cells in the dentate gyrus GCL (~2.6 fold p<0.001, n = 4) (Fig 7A and 7B) and hilus (~21 fold p<0.0001, n = 4) (Fig 7A and 7C) of CRAF^-/-^ compared to CRAF^+/+^ control mice. To positively determine the identity of the dying cells, we performed double immune labelling for activated caspase-3 and GFAP. Quantitative analysis of P30 brain serial sections (~20 sections per mouse) through the entire hippocampal dentate gyrus revealed that 88% of activated caspase-3 positive cells were also immunoreactive for GFAP (S6 Fig). Thus, the large majority of the dying cells, which are located at the inner granule cell layer, close to the subgranular zone, might derive from local radial glia cells.

## Discussion

In this report we investigate the role of the kinase CRAF in the postnatal murine brain development with a focus on alterations that occur in hippocampal neural precursor cells and their differentiation in vivo. CRAF knockout mice on an inbred background such as C57Bl/6 do not survive after birth [15, 16]. Here, we were able to overcome early postnatal death of CRAF-deficient mice by crossing the CRAF null allele for at least two generations with the outbred strain CD-1. In this genetic background, about one out of three CRAF-deficient animals lived for 30 or more days. About two out of three CRAF-deficient animals died within one day after birth, presumable due to massive apoptosis in lung and liver. Our results indicate that CRAF acts in a wide range of cells and tissues that can be modified by the genetic background. The molecular basis of these genetic modifiers that affect CRAF functions are not known yet. Identification of strain-specific modifier mechanisms might contribute to a better understanding of various functions of CRAF signalling.

Postnatal day P30 CRAF-deficient mice scored normally in a Rotarod task but displayed coordination problems of their limbs. In the brain of P10 and P30 mice, we detected shorter cerebellar lobules III and X in CRAF-deficient mice. One of the principal tasks of the cerebellum is to control motoric functions that occur without any recognition but need some feedback about the body position. The vestibule-cerebellum that concerns to LX [30] carries responsibility for the balance, whereas the spino-cerebellum that concerns to LI-V [30] has to control hold and support of motoric functions. Both structures are apparently affected by CRAF-deficiency in postnatal mice, both in functional as well as in morphological consideration. A similar observation was found in BRAF-deficient mice, indicating that BRAF as well as CRAF are involved in the proper development of this cerebellar structure [28].

The hippocampal granule cell volume of CRAF-deficient mice was not altered at P10 but was smaller at P30 indicating that CRAF plays an essential role in the development of the hippocampus beginning at an age of ten days in a phase where the generation of new granule neurons is a prominent feature of hippocampal development. The reduction in granule cell volume cannot be explained by reduction in the NPC proliferation, as we observed a significant increase in the numbers of actively dividing cells in the dentate gyrus subgranular zone and the hilus. We also identified that the dividing cells labelled by a 24h BrdU pulse were GFAP-positive and thus belong to the radial glial progenitor pool. To resolve the apparent discrepancy between increased proliferation of progenitor cells and reduced granule cell volume, we identified that there was a significant increase in the exit from the cell cycle in CRAF-deficient mice. CRAF-deficient cells prematurely exiting the cell cycle showed a reduced ability to generate NeuN-positive neurons six or twelve days after labelling of the progenitors with a single BrdU pulse. These cells apparently underwent apoptosis, as the number of apoptotic cells is increased in CRAF-deficient mice. Our findings may provide a mechanistic understanding of the defects seen in Noonan patients harbouring CRAF mutations with impaired kinase activity.

In adult hippocampal neurogenesis, new-born neurons become functionally integrated into the pre-existing trisynaptic circuit of the hippocampus: the new neurons form glutamatergic mossy fibre synapses with CA3 pyramidal neurons, and develop functional synapses with local interneurons [44, 45]. The extent of adult neurogenesis is regulated by physiological stimuli, such as enriched environment, task learning, physical activity and ageing [38, 46]. It depends on the activation of the progenitor cells from their quiescent state in G_0_ to divide and to subsequently differentiate into a granule neuron [38, 47]. Here, we observed an increased number of proliferating radial glia cells in the microenvironment of the neurogenic niche in the dentate gyrus, indicating that CRAF might be involved in the transit of progenitor cells from the quiescent state to the proliferative state. We found that CRAF deficiency resulted in an approximately 50% reduction in the number of new-born granule neurons 12 days after their generation. Therefore we assume that hippocampal functions that require new neurons are severely impaired in CRAF-deficient mice. These functions might include learning [48], anxiety-like behaviour [49], and spatial or episodic memory function [50–52]. To test whether these functions are really impaired in adult CRAF-deficient mice, experiments with a specific deletion of the CRaf gene from hippocampal neural progenitors would be needed.

The impact of alterations in RAF/MEK/ERK signaling in neurogenic regions has been the subject of several studies with genetically altered mice. In summary these studies reveal a complex pattern of functions of the diverse members of the RAF/MEK/ERK signalling pathway in the development of neurons and in postnatal neurogenic processes. In mice lacking BRAF expression in the brain, the size of the hippocampal granule cell layer is normal at postnatal day (P) 12 but diminished at P21. This defect was shown to be caused by a reduced ability of dentate gyrus progenitor cells to differentiate into NeuN-positive granule cell neurons [28]. *Mek2* deficient mice are viable, fertile and show no overt phenotypic alterations [53]. *Mek1* knockout mice were embryonic lethal [54] and show defects in vascularization of the placenta. In mice with *Mek2* null alleles and conditional inactivation of *Mek1* in the neural lineage no gross morphological abnormalities in the brain were observed at birth [55]. Loss of MEK expression in mice leads to a severe reduction of astrocyte and oligodendrocyte progenitors; however, the generation of new neurons at embryonic day 17.5 is not reduced in these animals [55]. Investigations on the roles of the terminal kinases in this signaling axis have revealed that constitutive inactivation of *Erk2* results in early embryonic lethality [56–58]. Mice with an *Erk1* loss-of function mutation are viable, fertile and of normal size, but show a severe defect in thymocyte maturation [59]. Notably, ablation of *Erk2* at mid neurogenesis using a hGFAP-Cre deleter mouse results in a reduction of intermediate progenitor cells as well as a reduced number of neurons in the forebrain [60]. Deleting *Erk2* at the onset of neurogenesis revealed a function for *Erk2* for developmental processes in radial glial neural progenitor cells. Inactivation of *Erk1* and *Erk2* leads to a reduction of both immature and mature granule neurons in the hippocampus at P10, presumably due to a loss of both intermediate progenitor and radial glial cells [27]. Apparently, the radial glial cells had converted into glial cells with horizontal processes at the expense of radial ones, unable to generate intermediate progenitor cells and granule neurons [27].

A potential mechanism underlying there alterations may be a weakened RAF kinase activity [61] due to the impossibility of BRAF/CRAF hetero-dimerization in response to RAF activation [12]. BRAF/BRAF homodimers or BRAF/ARAF heterodimers are well known to induce a weaker RAF kinase activity compared to BRAF/CRAF heterodimers [10, 62] and a compensation of CRAF by BRAF could be only noticed during the first half of gestation [63].

## Materials and methods

### Animals

All animal studies were performed in accordance with German legislation and were approved by the Bavarian State authorities for animal experimentation. All animal experiments were approved by the Committee on the Ethics of Animal Experiments of the Government of Lower Franconia (Permit Number: 55.2–2531.01-83/09). All surgery was performed under Ketanest/Rompun anesthesia [Ketamin (Ketasol) (100mg/ml 1:5 dilluted with sodium chloride) application: 2mg/30g body weight; Rompun (Xylazin) (20mg/ml 1:5 diluted with sodium chloride) application 0.4mg/30g body weight], and all efforts were made to minimize suffering. Mice were housed under barrier condition in air-filtered and temperature-controlled animal facility with a 12h light/dark cycle and free access to food and water. Animals were daily monitored for signs of distress. Heterozygous (129Ola/C57Bl/6) CRAF ko mice were kindly provided by Catrin Pritchard who generated a null-allele by the deletion of exon 10 to exon 13 [17]. We outcrossed these mice to a CD1 outbred background (Charles River), so all of our mice were of similar genetic background (≥75% CD1) and CRAF-deficient littermates were generated by heterozygous intercrosses. Altogether we analyzed n = 2778 mice. Genotype analysis was performed by PCR as described earlier [17]. The primer pair for detection of the wild-type allele (wt) was A (5'ACA GAA AGT GTA GCT GCA GTG A) and B (5' ATT GAT TTG ATT GCC AGG TAT GAT) yielding a fragment of 335bp. To detect the CRAF ko allele primer A (5'ACA GAA AGT GTA GCT GCA GTG A) was combined with primer C (5' TGC GTG CAA TCC ATC TTG TTC AA) to achieve a PCR-fragment of 450bp.

### Western blot

For Western blot analysis, tissue was homogenized in 50 mM Tris-HCl (pH8.0), 100mM NaCl, 10mM EDTA, 0.5% NP-40 and protease inhibitors (Sigma). Tissue lysates were cleared by centrifugation and solubilized protein was separated by SDS-PAGE. Proteins were blotted on nitrocellulose membranes (PROTRAN, Whatman). The following antibodies were used: rabbit anti-CRAF (Santa Cruz, sc-227) rabbit anti-phospho-ERK1/2 (Cell Signalling, #9101) and rabbit anti-β-actin (Santa Cruz, sc-1616-R). For detection, the ECL kit (GE Healthcare) was used in combination with horseradish peroxidase-coupled secondary antibodies (Dianova).

### BrdU labelling and histological analysis

Bromodeoxyuridine (BrdU) (Sigma #B9285-1G) application (intraperitoneally, 50μg/g body weight in 0.9% sodium chloride) was performed to permanent label proliferating S-phase cells [32, 64, 65]. Mice were anaesthetized with Ketanest (2mg/30g body weight)/Rompun (0.4mg/30g body weight) and transcardially perfused with saline (pH7.4) to remove the blood and finally kill the mice, followed by 4% paraformaldehyde (PFA) in PBS (pH7.4) perfusion for fixation. Tissue were dissected and postfixed overnight in 4% PFA at 4°C. Brains were separated into left and right hemisphere whereby the latter was transferred into 30% sucrose in PBS (pH7.4) after PBS washing, OCT-embedded and cryosected (Leica CM1900). The left part was always paraffin-embedded and cut with a slide microtome (Leica RM2155). Both hemispheres were serially sectioned into a spate of ten 10 μm consecutive sagittal sections on Superfrost plus (Roth) slides to analyze single cells with various differentiation markers as described in [65]. We always used the first slide for Nissl staining from the beginning of the DG-formation until its fusion. Quantified data were later normalized to the Nissl-stained DG granule cell layer (GCL) volume [32], summarized and multiplied with the slide thickness as described in [65]. Data show counted cells per volume in mm^3^. Analysis have been performed using Keyence 8000 measuring software with a 20xPlan APO or 40xPlan APO Nikon Objective. Nissl stained cerebellum sections were pictured by a Nikon photo camera in combination with a stereomicroscope (Leica).

### Immunohistochemistry

Immuno-histological and immunofluorescent analysis were performed on frozen (cryo) or paraffin-embedded 10 μm serial sections. After deparaffinization, sections were carefully boiled in a microwave oven in 10 mM citrate buffer pH 6.0 for 10 minutes (23 minutes for Ki67 staining). For anti-BrdU-labelling, antigen-retrieval was performed in 2N HCl for 30 min at 60°C. Then, slices were treated with 0.1 M sodium borate buffer pH 8.5 for 25 min at room temperature (RT). Antibodies against the following proteins were used (o/N 4°C): rat anti-BrdU (Abcam ab6326, 1:100), sheep anti-BrdU (Abcam ab1893, 1:128), mouse anti-calbindin (Sigma C9848, 1:1.000), rabbit anti-activated caspase 3 (Cell Signaling, #9661, 1:100), mouse anti-CRAF (BD, clone 53, 1:100 and Santa Cruz, sc-7267, 1:100), rabbit anti-CRAF (Santa Cruz sc-227, 1:100), rabbit anti-GFAP (Dako, Z0334, 1:200), chicken anti-GFAP (Abcam, ab4674, 1:200), rabbit anti-Ki67 (Thermo Scientific SP6, 1:200), chicken anti-Map2 (Abcam ab5392, 1:300), rabbit anti-MAP2 (Abcam ab32454, 1:200), mouse anti-NeuN (Millipore MAB337, 1:100 or MAB337B, 1:100), and mouse anti-Nestin (DSHB rat-401, 1:5). The following secondary antibodies were used: goat anti-rat Alexa 488 (Invitrogen, 1:200), rabbit anti-sheep FITC (Abcam, 1:200), donkey anti-rabbit Cy3 (Dianova, 1:400), goat anti-chicken Alexa 488 (Invitrogen, A11039, 1:600), biotinylated goat anti-rabbit IgG (Dako, 1:200), rabbit anti-mouse IgG (Dako, 1:400), streptavidin Alexa555 (Invitrogen, 1:200) or Texas Red Streptavidin (Vector, 1:200). DAPI (1:9000) was used to label cell nuclei. CRAF and activated caspase 3 staining were performed with biotinylated anti-rabbit in combination with ABC (advanced avidin/biotin technology) (Vector, 1:50) and visualized with 3,3`-Diaminobenzidin (DAB) (Sigma) [65].

For epifluorescence microscopy a Keyence Biozero 8000 microscope was used in combination with a Plan Fluor 20x, 0.50 NA objective (Nikon). Confocal images were either acquired using an inverted IX81 microscope equipped with an Olympus FV1000 confocal laser scanning system, a FVD10 SPD spectral detector and diode lasers of 405, 473, 559, and 635 nm. Images were acquired with an Olympus UPLSAPO40x (oil, numerical aperture: 1.3) objective, or a confocal Leica SP5 microscope equipped with a 40x 1.25–0.75 oil objective.

### Mouse behavioral analysis

Behavior analysis on motor coordination was conducted with acceleration from 4 to 40 r.p.m. in a Rotarod 7650 device (Ugo Basile) as described in [66]. Postnatal P30 day old male CRAF^-/-^ (CRAF ko) and CRAF^+/+^ control mice (n = 3) were placed on the Rotarod device and the time was counted until the mouse fell from the Rotarod [66] or after a maximum time of 300s was recorded [66]. Each mouse was tested three times after one test run with an adequate period of recovery between each run. Results were given as the average time that each group (CRAF ko and control) was able to keep up balanced on the Rotarod [66].

Additionally we analyzed motor coordination by walking and balancing on a pencil as described in [65]. Analysis were performed with CRAF^-/-^ and control male mice at postnatal day P30 (n = 3). We counted the time spent by balancing on the pencil with a cut-off time after 60s [65]. Test subjects that fall down from the pencil immediately obtained three further trials. All CRAF^-/-^ mice fall down between one to two seconds whereas control mice were able to balance without any problem for even longer time frames as already reported in [65].

### Quantification and statistical analysis

BrdU labelled nuclei were counted in a spate of ten serial sections through one hemisphere analogous to [67]. The total number of analyzed cells was summarized and divided through the measured Nissl volume (cells/mm^3^). Double-immunofluorescence labelling was analyzed by z-stack microscopy method using 40x Plan APO optical view (Keyence BZ 8000). Data are presented as mean ± s.e.m. Paired t-test (two-tailed) was used to compare two groups (of siblings) and p<0.05 was considered significant. Data values in each group were assessed for normal distribution using Statistica 8.0 (Statsoft) quantile plot test. Data sets for non- normal distribution were analyzed with Wilcoxon test for paired sets.

## Supporting information

S1 Fig Phenotypic observations in newborn and postnatal CRAF ko mice. (A) Quantitative analysis of new born CRAF ko (white bar) mice at P0/P1 with an eye-open-at-birth (EOB) phenotype shown as a fraction of all analysed new born CRAF ko mice. Data are mean; P0/P1, n>10.
(B) Quantitative analysis of postnatal CRAF ko mice with an eyelid closure defect at P30. 50% of CRAF ko at P30 show a both eyelid closed phenotype (grey bar), whereas 42.5% of CRAF ko at P30 have one eyelid closed (white bar) and 7.5% of CRAF ko at P30 show a normal eyelid phenotype with both eyes open (dark bar). Data are mean; P30, n = 40.
(C) Body weight increase of postnatal CRAF ct (dark bars) and CRAF ko (white bars) mice from P23 until P34 during the BrdU long-chase experiment. Data are shown as mean ± s.e.m.; n = 6. Significant differences are shown in p-value as indicated.
(TIF)

S2 Fig Behaviour analysis concerning motoric function on a Rotarod in postnatal CRAF ko mice. (A) Loss of motoric coordination of front and hind limbs in postnatal CRAF ko mice at P30 leads to a loss in catching the cage top with the hind limbs. Without the support of hind limbs, CRAF ko mice cannot reach the cage top and fall down immediately (inlay), whereas control mice (left) can hang down head without any impairment (n = 3).
(B) Impaired ability to balance on a small rod. CRAF ko mice fall down immediately (<1 sec.), whereas CRAF ct mice (left) can move from left to right without any impairment in changing their body orientation (inlays) (n = 3).
(C) Representative images of CRAF ct (left) and CRAF ko (right) mice on an accelerating Rotarod at P30 (n = 3). CRAF ko (right) mice do not show any general impaired motoric function moving on a Rotarod.
(D) Quantitative analysis of running time on a Rotarod. CRAF ct mice (black bar), CRAF mice (white bar). Data are mean ± s.e.m.; n = 3, P30. No significant differences could be detected.
(TIF)

S3 Fig Microscopic analysis of sagittal Nissl stained brain sections of postnatal CRAF ko and control mice at postnatal day P10 and P30. (A) Representative images of CRAF ct (left) and CRAF ko (right) sagittal brain sections stained for Nissl at postnatal day P10. No general morphological alteration was observed with the exception of the cerebellum of CRAF ko (white arrowhead). Scale bar 100μm.
(B) Representative images of CRAF ct (left) and CRAF ko (right) sagittal brain sections stained for Nissl at postnatal day P30. No general morphological alteration was observed with the exception of the cerebellum of CRAF ko (white arrowhead). Scale bar 100μm.
(TIF)

S4 Fig CRAF-deficiency in the cerebellum of postnatal mice. (A) Immune-histological analysis of CRAF (brown) expression in the cerebellum of sagittal brain sections of postnatal CRAF ct (left) and CRAF ko (right) mice at P10. Representative sections of lobule (L) X of CRAF ko exhibit any positive CRAF expression in the cerebellar Purkinje cells (right, white arrowheads) compared to CRAF ct (left, white arrowheads). Scale bar = 50μm.
(B) Immune-histological analysis of CRAF (brown) expression in the cerebellum of sagittal brain sections of postnatal CRAF ct (left) and CRAF ko (right) mice at P30. Representative sections of lobule (L) X of CRAF ko exhibit any positive CRAF expression in the cerebellar Purkinje cells (right, white arrowheads) compared to CRAF ct (left, white arrowheads). Scale bar = 50μm.
(C) Representative sagittal brain sections of P30 CRAF ct sections of hippocampus (left) and cerebellum (right) stained with secondary antibody only to visualize unspecific background staining. Scale bar = 50μm.
(TIF)

S5 Fig Increased numbers of BrdU^+^/GFAP^+^ radial astrocytes (rA) compared to BrdU^+^/GFAP^+^ horizontal astrocytes (hA) in the DG GCL of CRAF ko at P34 12 days after a single BrdU application. (A) BrdU/GFAP positive radial astrocytes (rA) as a fraction of BrdU-labelled cells in the dentate gyrus (DG) GCL of CRAF ct (dark bar) and CRAF ko (white bar) at P35 (n = 6) 12 days after a single BrdU application. Data are mean ± s.e.m.; significant differences are shown in p-value p = 0.0009.
(B) BrdU/GFAP positive horizontal astrocytes (hA) as a fraction of BrdU-labelled cells in the dentate gyrus (DG) GCL of CRAF ct (dark bar) and CRAF ko (white bar) at P35 (n = 6) 12 days after a single BrdU application. Data are mean ± s.e.m.; significant differences are shown in p-value p = 0.0006.
(C) BrdU/GFAP positive rA and hA of CRAF ct (dark bar) and CRAF ko (white bar) at P35 (n = 6) 12 days after a single BrdU application as a fraction of BrdU-labelled cells in the dentate gyrus (DG) GCL. Data are mean ± s.e.m.; significant differences are shown in p-value CRAF ct rA/hA p<0.0001; CRAF ko rA/hA p<0.0001.
(TIF)

S6 Fig Identification of the increased population of caspase-3 positive cells in the dentate gyrus GCL of CRAF^-/-^ mice. (A) Double immune labelling of activated caspase 3 (dark) and GFAP (red) in CRAF^-/-^ dentate gyrus (n = 3) at the age of P30. Dark arrowheads indicate activated caspase 3 positive cells that colocalize with GFAP (merge) in the inner granule cell layer, close to the subgranular zone.
(B) Quantitative analysis of activated caspase 3 positive cells in the dentate gyrus (DG) GCL of CRAF ko (white bar) that colocalize with GFAP. Caspase 3/GFAP-positive cells are mean ± s.e.m.; P30, n = 3 and were shown as a fraction of the total number of caspase 3 positive cells. Serial sections were analysed (~20/animal) from the entire hippocampal dentate gyrus.
(TIF)

S7 Fig Western Blot analysis of CRAF^-/-^ and control lysates concerning ERK1/2 phosphorylation. A) Western Blot analysis of CRAF control (CRAF^+/+^, left) and CRAF ko (CRAF^-/-^, right) lysates of CB (cerebellum), HP (hippocampus), PC (prefrontal cortex) and OB (olfactory bulb) at the age of P30. No differences in the pERK1/2 expression could be observed. Actin serves as loading control.
(TIF)
