# Supplementary figures and images for: Impaired neuronal maturation of hippocampal neural progenitor cells in mice lacking CRAF
Source: PLoS One. 2018 Mar 28;13(3):e0192067. doi: 10.1371/journal.pone.0192067 (PMC5873938; doi:10.1371/journal.pone.0192067)

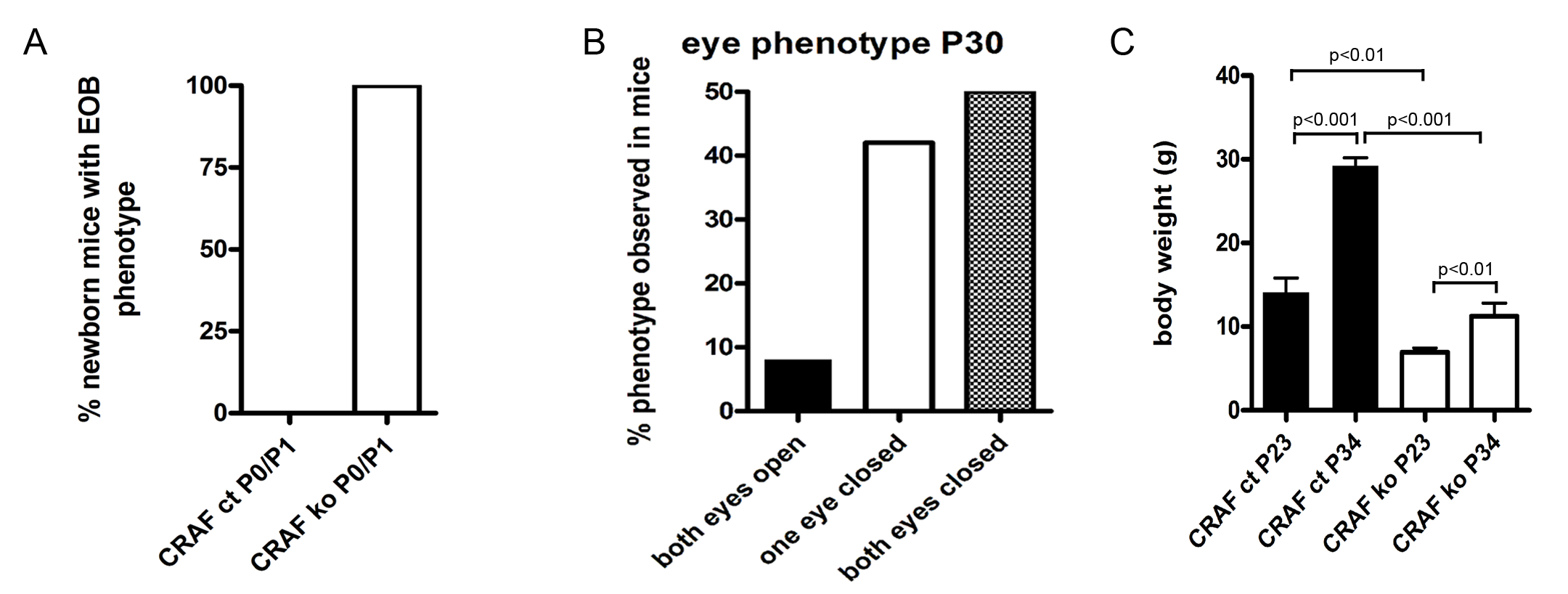

Supplement: S1 Fig — (A) Quantitative analysis of new born CRAF ko (white bar) mice at P0/P1 with an eye-open-at-birth (EOB) phenotype shown as a fraction of all analysed new born CRAF ko mice. Data are mean; P0/P1, n>10. (B) Quantitative analysis of postnatal CRAF ko mice with an eyelid closure defect at P30. 50% of CRAF ko at P30 show a both eyelid closed phenotype (grey bar), whereas 42.5% of CRAF ko at P30 have one eyelid closed (white bar) and 7.5% of CRAF ko at P30 show a normal eyelid phenotype with both eyes open (dark bar). Data are mean; P30, n = 40. (C) Body weight increase of postnatal CRAF ct (dark bars) and CRAF ko (white bars) mice from P23 until P34 during the BrdU long-chase experiment. Data are shown as mean ± s.e.m.; n = 6. Significant differences are shown in p-value as indicated. (TIF) [file pone.0192067.s001.tif]

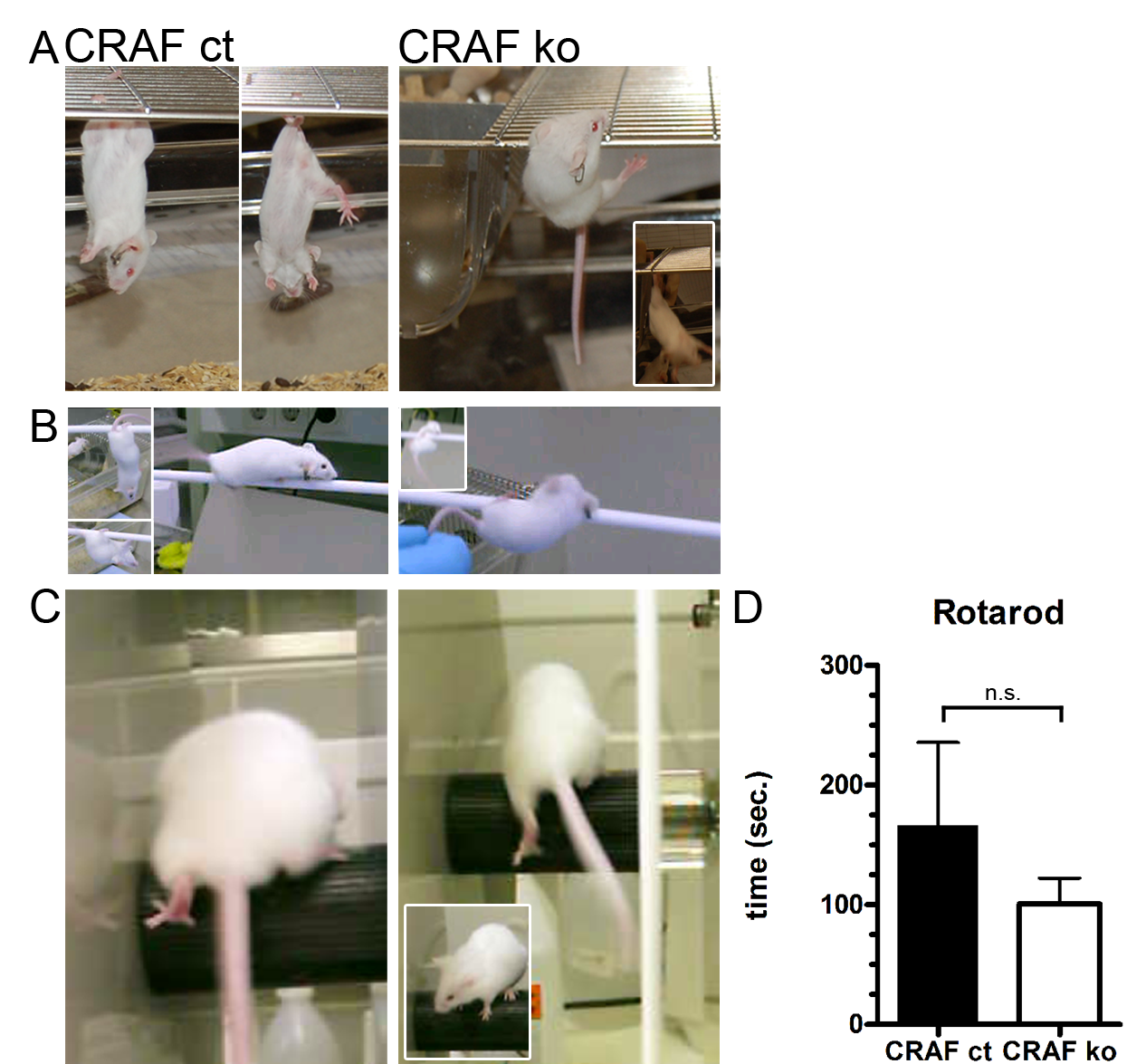

Supplement: S2 Fig — (A) Loss of motoric coordination of front and hind limbs in postnatal CRAF ko mice at P30 leads to a loss in catching the cage top with the hind limbs. Without the support of hind limbs, CRAF ko mice cannot reach the cage top and fall down immediately (inlay), whereas control mice (left) can hang down head without any impairment (n = 3). (B) Impaired ability to balance on a small rod. CRAF ko mice fall down immediately (<1 sec.), whereas CRAF ct mice (left) can move from left to right without any impairment in changing their body orientation (inlays) (n = 3). (C) Representative images of CRAF ct (left) and CRAF ko (right) mice on an accelerating Rotarod at P30 (n = 3). CRAF ko (right) mice do not show any general impaired motoric function moving on a Rotarod. (D) Quantitative analysis of running time on a Rotarod. CRAF ct mice (black bar), CRAF mice (white bar). Data are mean ± s.e.m.; n = 3, P30. No significant differences could be detected. (TIF) [file pone.0192067.s002.tif]

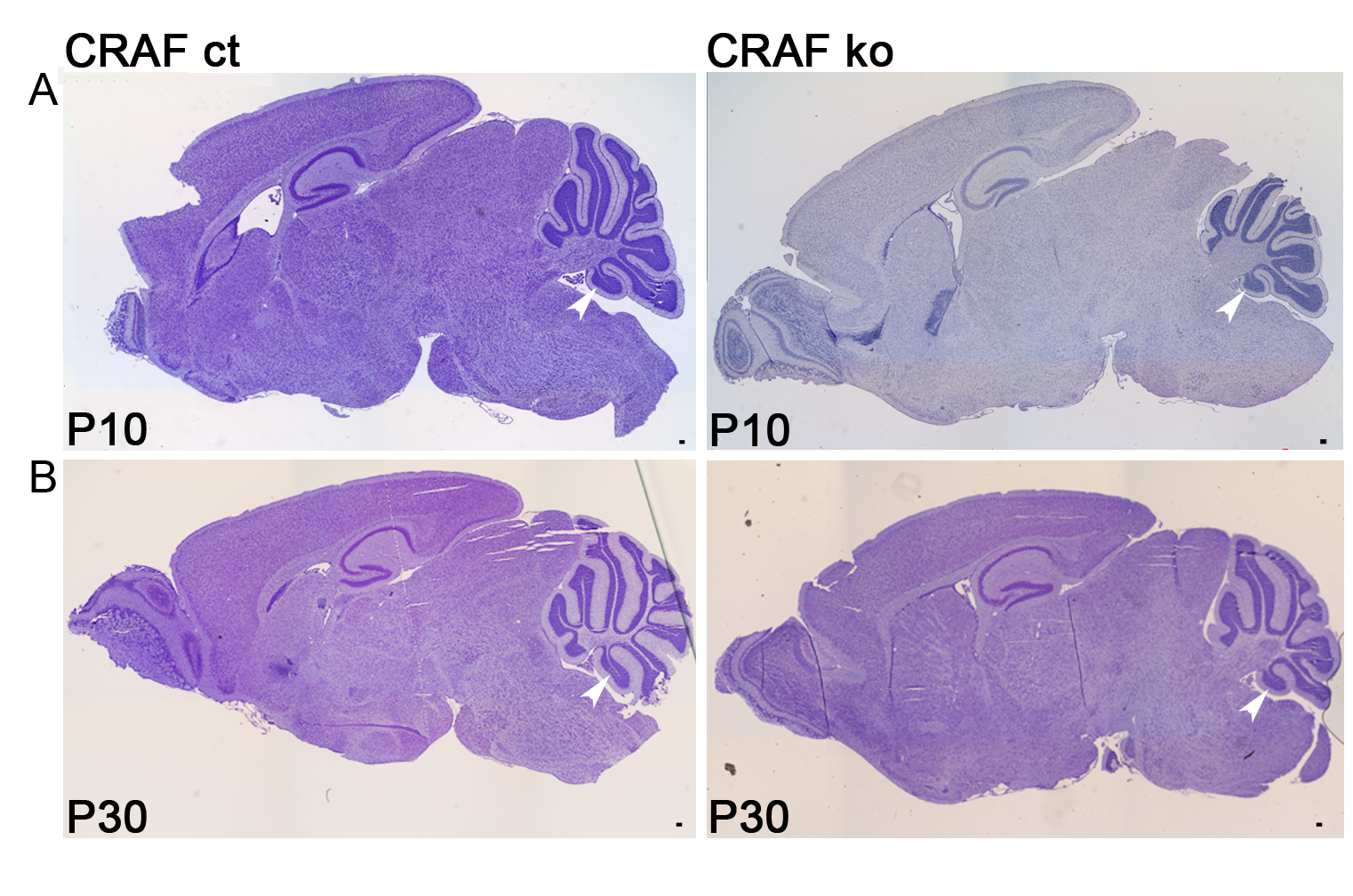

Supplement: S3 Fig — (A) Representative images of CRAF ct (left) and CRAF ko (right) sagittal brain sections stained for Nissl at postnatal day P10. No general morphological alteration was observed with the exception of the cerebellum of CRAF ko (white arrowhead). Scale bar 100μm. (B) Representative images of CRAF ct (left) and CRAF ko (right) sagittal brain sections stained for Nissl at postnatal day P30. No general morphological alteration was observed with the exception of the cerebellum of CRAF ko (white arrowhead). Scale bar 100μm. (TIF) [file pone.0192067.s003.tif]

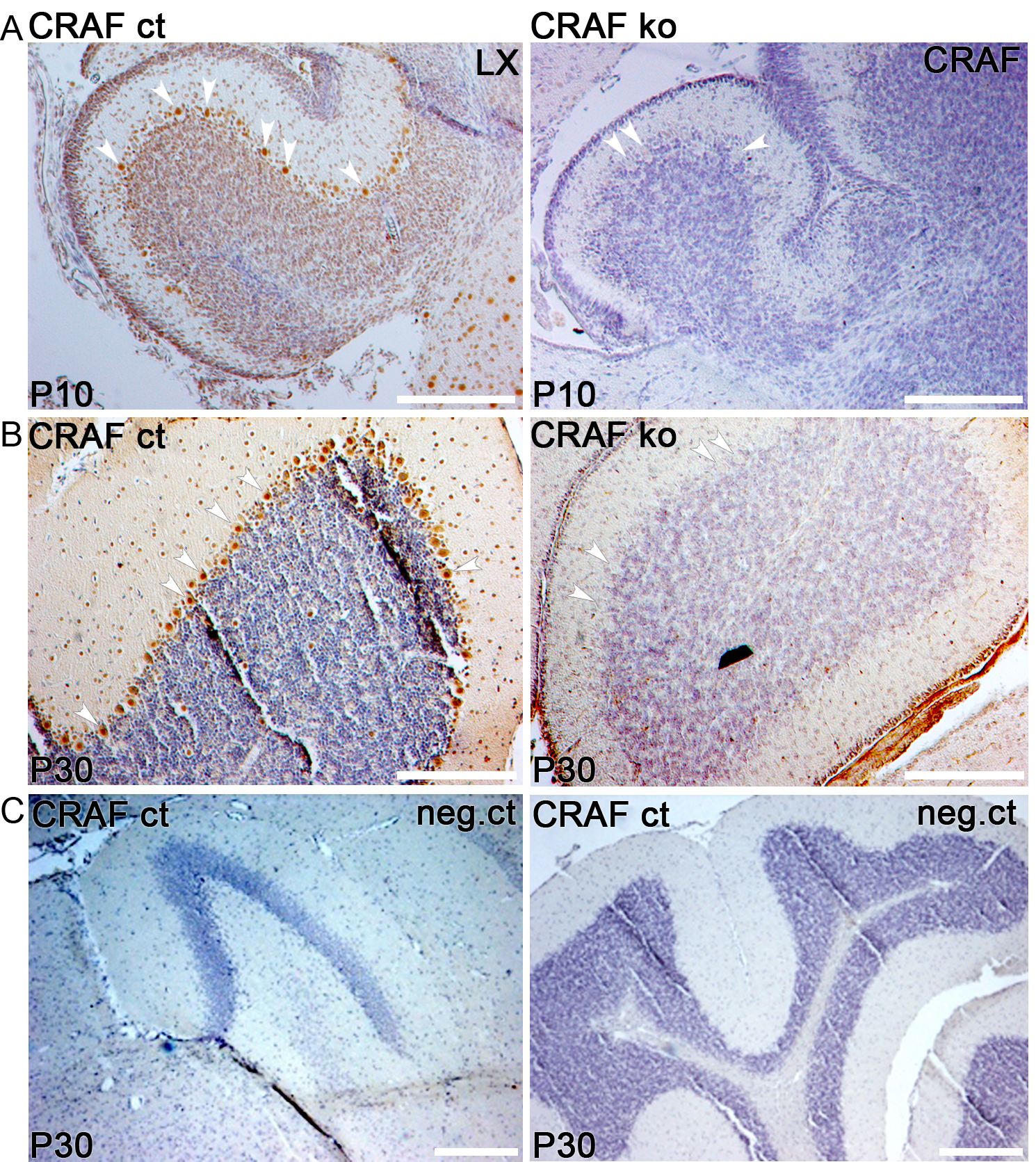

Supplement: S4 Fig — (A) Immune-histological analysis of CRAF (brown) expression in the cerebellum of sagittal brain sections of postnatal CRAF ct (left) and CRAF ko (right) mice at P10. Representative sections of lobule (L) X of CRAF ko exhibit any positive CRAF expression in the cerebellar Purkinje cells (right, white arrowheads) compared to CRAF ct (left, white arrowheads). Scale bar = 50μm. (B) Immune-histological analysis of CRAF (brown) expression in the cerebellum of sagittal brain sections of postnatal CRAF ct (left) and CRAF ko (right) mice at P30. Representative sections of lobule (L) X of CRAF ko exhibit any positive CRAF expression in the cerebellar Purkinje cells (right, white arrowheads) compared to CRAF ct (left, white arrowheads). Scale bar = 50μm. (C) Representative sagittal brain sections of P30 CRAF ct sections of hippocampus (left) and cerebellum (right) stained with secondary antibody only to visualize unspecific background staining. Scale bar = 50μm. (TIF) [file pone.0192067.s004.tif]

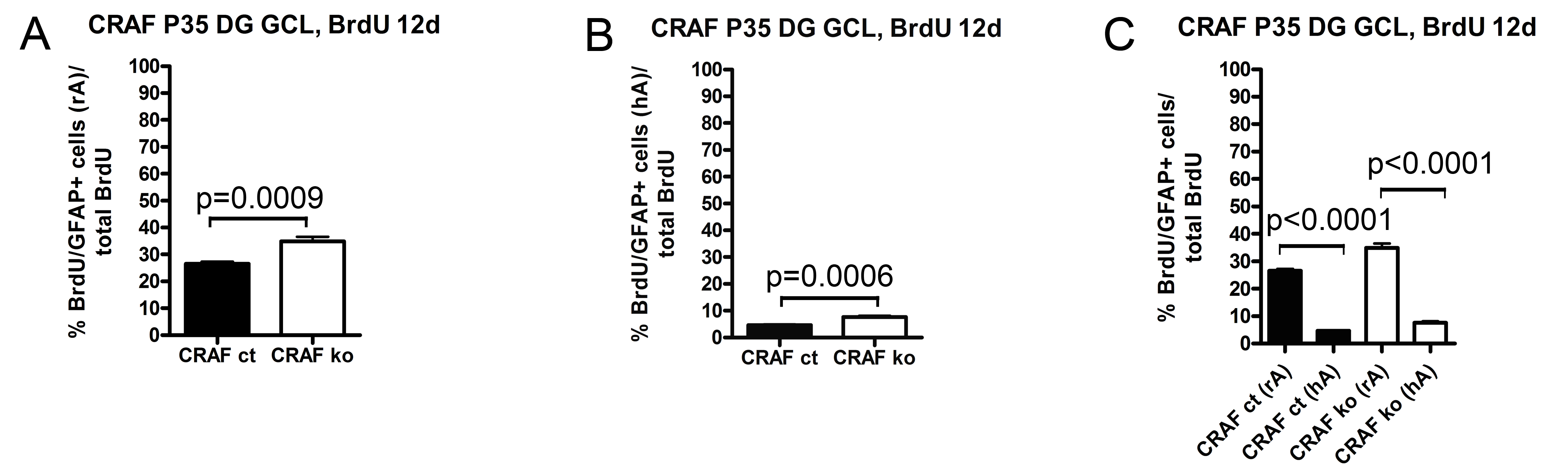

Supplement: S5 Fig — (A) BrdU/GFAP positive radial astrocytes (rA) as a fraction of BrdU-labelled cells in the dentate gyrus (DG) GCL of CRAF ct (dark bar) and CRAF ko (white bar) at P35 (n = 6) 12 days after a single BrdU application. Data are mean ± s.e.m.; significant differences are shown in p-value p = 0.0009. (B) BrdU/GFAP positive horizontal astrocytes (hA) as a fraction of BrdU-labelled cells in the dentate gyrus (DG) GCL of CRAF ct (dark bar) and CRAF ko (white bar) at P35 (n = 6) 12 days after a single BrdU application. Data are mean ± s.e.m.; significant differences are shown in p-value p = 0.0006. (C) BrdU/GFAP positive rA and hA of CRAF ct (dark bar) and CRAF ko (white bar) at P35 (n = 6) 12 days after a single BrdU application as a fraction of BrdU-labelled cells in the dentate gyrus (DG) GCL. Data are mean ± s.e.m.; significant differences are shown in p-value CRAF ct rA/hA p<0.0001; CRAF ko rA/hA p<0.0001. (TIF) [file pone.0192067.s005.tif]

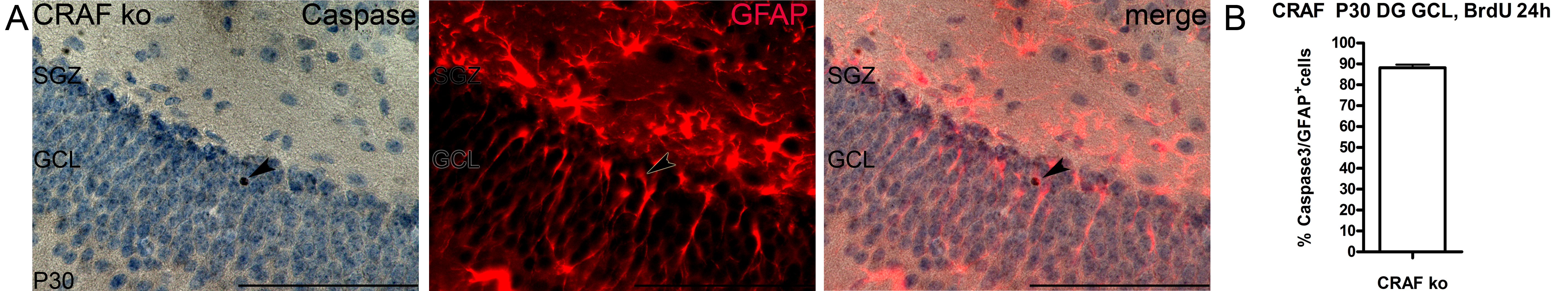

Supplement: S6 Fig — (A) Double immune labelling of activated caspase 3 (dark) and GFAP (red) in CRAF-/- dentate gyrus (n = 3) at the age of P30. Dark arrowheads indicate activated caspase 3 positive cells that colocalize with GFAP (merge) in the inner granule cell layer, close to the subgranular zone. (B) Quantitative analysis of activated caspase 3 positive cells in the dentate gyrus (DG) GCL of CRAF ko (white bar) that colocalize with GFAP. Caspase 3/GFAP-positive cells are mean ± s.e.m.; P30, n = 3 and were shown as a fraction of the total number of caspase 3 positive cells. Serial sections were analysed (~20/animal) from the entire hippocampal dentate gyrus. (TIF) [file pone.0192067.s006.tif]

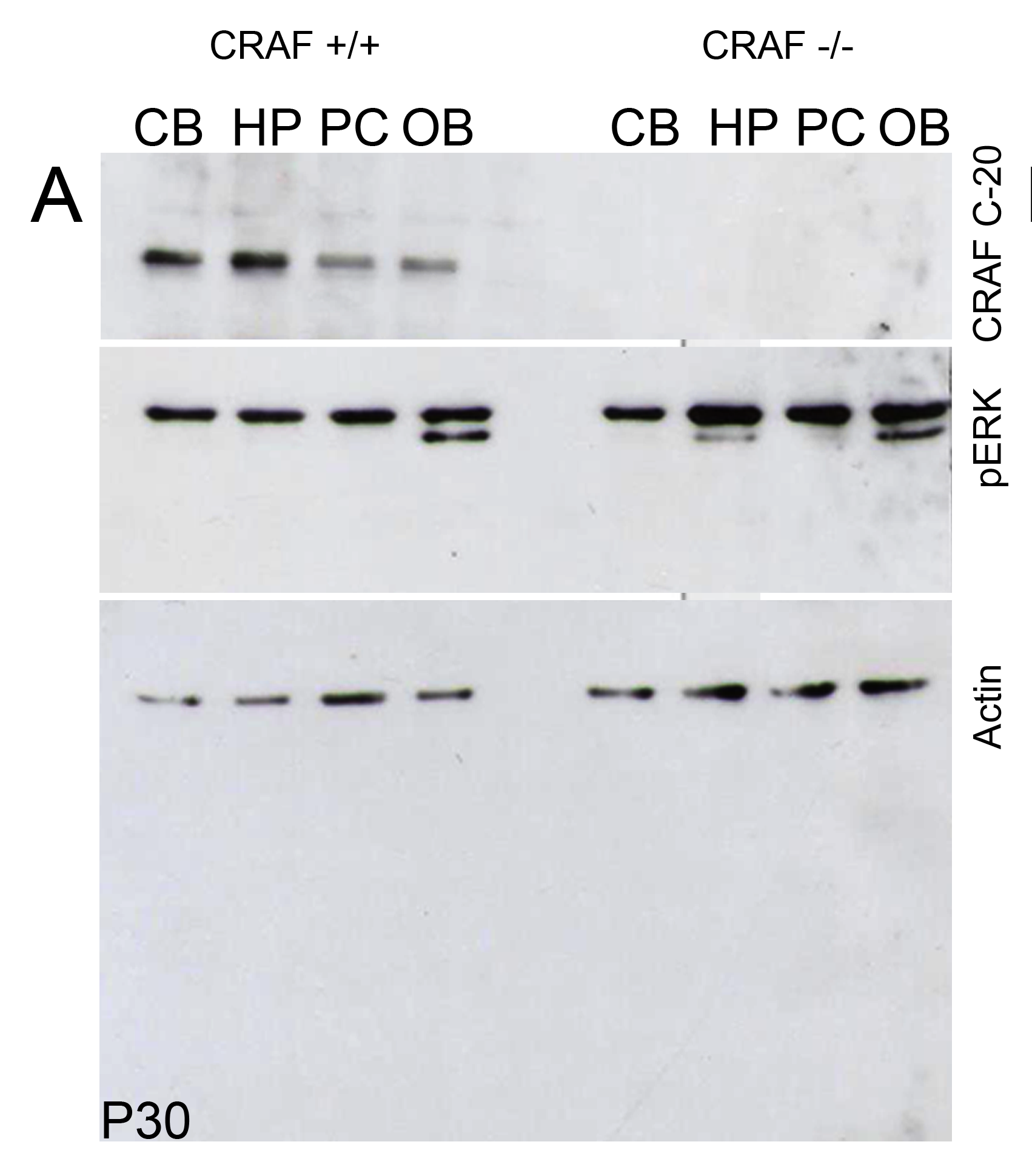

Supplement: S7 Fig — A) Western Blot analysis of CRAF control (CRAF+/+, left) and CRAF ko (CRAF-/-, right) lysates of CB (cerebellum), HP (hippocampus), PC (prefrontal cortex) and OB (olfactory bulb) at the age of P30. No differences in the pERK1/2 expression could be observed. Actin serves as loading control. (TIF) [file pone.0192067.s007.tif]
